# *SCN1A* overexpression, associated with a genomic region marked by a risk variant for a common epilepsy, raises seizure susceptibility

**DOI:** 10.1007/s00401-022-02429-0

**Published:** 2022-05-12

**Authors:** Katri Silvennoinen, Kinga Gawel, Despina Tsortouktzidis, Julika Pitsch, Saud Alhusaini, Karen M. J. van Loo, Richard Picardo, Zuzanna Michalak, Susanna Pagni, Helena Martins Custodio, James Mills, Christopher D. Whelan, Greig I. de Zubicaray, Katie L. McMahon, Wietske van der Ent, Karolina J. Kirstein-Smardzewska, Ettore Tiraboschi, Jonathan M. Mudge, Adam Frankish, Maria Thom, Margaret J. Wright, Paul M. Thompson, Susanne Schoch, Albert J. Becker, Camila V. Esguerra, Sanjay M. Sisodiya

**Affiliations:** 1 Department of Clinical and Experimental Epilepsy, UCL Queen Square Institute of Neurology, Box 29, Queen Square, London, WC1N 3BG UK; 2 Chalfont Centre for Epilepsy, Chalfont St Peter, Bucks, SL9 0RJ UK; 3 Chemical Neuroscience Group, Centre for Molecular Medicine Norway (NCMM), Faculty of Medicine, University of Oslo, 0349 Oslo, Norway; 4 Department of Experimental and Clinical Pharmacology, Medical University of Lublin, 20-090 Lublin, Poland; 5 Institute of Neuropathology, Medical Faculty, University of Bonn, Section for Translational Epilepsy Research, 53127 Bonn, Germany; 6 Department of Epileptology, Medical Faculty, University of Bonn, 53127 Bonn, Germany; 7 Department of Molecular and Cellular Therapeutics, The Royal College of Surgeons in Ireland, Dublin 2, Ireland; 8 Department of Neurology, Yale University School of Medicine, New Haven, CT 06520 USA; 9 Department of Epileptology and Neurology, RWTH Aachen University, 52074 Aachen, Germany; 10 Department of Neuropathology, UCL Queen Square Institute of Neurology, London, WC1N 3BG UK; 11 Imaging Genetics Center, Mark and Mary Stevens Neuroimaging and Informatics Institute, Keck School of Medicine, University of Southern California, Marina del Rey, Los Angele, CA 90292 USA; 12 School of Psychology, Faculty of Health, Queensland University of Technology (QUT), Brisbane, QLD 4059 Australia; 13 School of Clinical Sciences, Faculty of Health, Queensland University of Technology (QUT), Brisbane, QLD 4029 Australia; 14 European Molecular Biology Laboratory, European Bioinformatics Institute, Wellcome Genome Campus, Cambridge, CB10 1SD UK; 15 Queensland Brain Institute, University of Queensland, St Lucia, 4072 QLD Australia

**Keywords:** Febrile seizures, Hippocampal sclerosis, Zebrafish, MRI, Genotype

## Abstract

Mesial temporal lobe epilepsy with hippocampal sclerosis and a history of febrile seizures is associated with common variation at rs7587026, located in the promoter region of *SCN1A.* We sought to explore possible underlying mechanisms. *SCN1A* expression was analysed in hippocampal biopsy specimens of individuals with mesial temporal lobe epilepsy with hippocampal sclerosis who underwent surgical treatment, and hippocampal neuronal cell loss was quantitatively assessed using immunohistochemistry. In healthy individuals, hippocampal volume was measured using MRI. Analyses were performed stratified by rs7587026 type. To study the functional consequences of increased *SCN1A* expression, we generated, using transposon-mediated bacterial artificial chromosome transgenesis, a zebrafish line expressing exogenous *scn1a*, and performed EEG analysis on larval optic tecta at 4 day post-fertilization. Finally, we used an in vitro promoter analysis to study whether the genetic motif containing rs7587026 influences promoter activity. Hippocampal *SCN1A* expression differed by rs7587026 genotype (Kruskal–Wallis test *P* = 0.004). Individuals homozygous for the minor allele showed significantly increased expression compared to those homozygous for the major allele (Dunn’s test *P* = 0.003), and to heterozygotes (Dunn’s test *P* = 0.035). No statistically significant differences in hippocampal neuronal cell loss were observed between the three genotypes. Among 597 healthy participants, individuals homozygous for the minor allele at rs7587026 displayed significantly reduced mean hippocampal volume compared to major allele homozygotes (Cohen’s *D* = − 0.28, *P* = 0.02), and to heterozygotes (Cohen’s *D* = − 0.36, *P* = 0.009). Compared to wild type, *scn1lab*-overexpressing zebrafish larvae exhibited more frequent spontaneous seizures [one-way ANOVA *F*(4,54) = 6.95 (*P* < 0.001)]. The number of EEG discharges correlated with the level of *scn1lab* overexpression [one-way ANOVA *F*(4,15) = 10.75 (*P* < 0.001]. Finally, we showed that a 50 bp promoter motif containing rs7587026 exerts a strong regulatory role on *SCN1A* expression, though we could not directly link this to rs7587026 itself. Our results develop the mechanistic link between rs7587026 and mesial temporal lobe epilepsy with hippocampal sclerosis and a history of febrile seizures. Furthermore, we propose that quantitative precision may be important when increasing *SCN1A* expression in current strategies aiming to treat seizures in conditions involving *SCN1A* haploinsufficiency, such as Dravet syndrome.

**Supplementary Information:**

The online version contains supplementary material available at 10.1007/s00401-022-02429-0.

## Introduction

The epilepsies are a set of conditions defined by the occurrence of seizures, often associated with comorbidities, impaired quality of life and shortened lifespan [56]. In many epilepsies, there is evidence of risk susceptibility imparted by common genetic variation [25, 35], but the mechanistic link between genetic variation and phenotype is typically obscure [25]. Increasingly, rare variants are being shown to underlie many of the rare epilepsies, especially the early onset conditions called the developmental and epileptic encephalopathies. *SCN1A* is amongst the best studied genes in epilepsy: rare pathogenic variants cause a variety of types of epilepsy, often characterised by seizures provoked by fever, as seen in Dravet syndrome [9]. Variants leading to Dravet syndrome cause loss of function, and are typically de novo in origin [26]. *SCN1A* variants may also cause familial epilepsies, such as genetic epilepsy with febrile seizures plus (GEFS +) [64, 65]. Moreover, we showed an association between common variation in *SCN1A* and the distinct epilepsy syndrome of mesial temporal lobe epilepsy with hippocampal sclerosis with history of febrile seizures (MTLEHS + FS) [29]. Despite its location overlapping with promoter regions of *SCN1A*, we were previously unable to demonstrate an association between the most strongly linked single nucleotide polymorphism (SNP; rs7587026) and expression of *SCN1A* in therapeutically resected human lateral temporal neocortex [29]. We hypothesized that minor allele homozygosity would influence *SCN1A* expression levels in the hippocampus with consequences linked to MTLEHS + FS.

Through a combination of expression data from affected human tissue, an MRI study of the impact of this variant on hippocampal size in healthy humans and functional data from an experimental model, we show that rs7587026 is associated with increased expression of *SCN1A* in human hippocampal tissue, which in turn reduces human hippocampal volume in healthy controls. Increased *Scn1a* expression lowers seizure threshold in an animal model. Finally, using in silico and in vitro promoter analysis, we demonstrate that rs7587026 lies in a highly constrained region and that the rs7587026-containing genomic motif exerts a strong regulatory role on *SCN1A* expression. We thus reveal a possible path between common genetic variation and a common human epilepsy (MTLEHS + FS).

## Materials and methods

### *SCN1A* expression and neuronal loss in hippocampal specimens from individuals with mesial temporal lobe epilepsy with hippocampal sclerosis

We analysed *SCN1A* mRNA expression in all available hippocampal biopsy specimens from 91 patients (44 female; aged 4–65 years) with pharmacoresistant mesial temporal lobe epilepsy with hippocampal sclerosis (MTLEHS) who had undergone surgical treatment at the University of Bonn Medical Centre between 1998 and 2008. Case ascertainment and clinical characteristics have been detailed previously [33, 43, 44]. All procedures were conducted in accordance with the Declaration of Helsinki. This study was approved by the Ethical Commission of University Hospital Bonn (222/16). Informed written consent was obtained from every patient. We adhered to the legal provisions governing the handling of personal data.

For all patients, genotyping was performed from surgical tissue for rs7587026. Typing was also performed for rs922224, another intronic SNP around *SCN1A* previously associated with MTLEHS + FS [29], unlinked to rs7587026 (*r*^2^ < 0.2), and rs6432860, an *SCN1A* SNP associated with febrile seizures (FS) only [15]. To examine possible allele-specific differences in expression levels, we compared *SCN1A* mRNA expression in hippocampal biopsy specimens by SNP types.

#### Sample preparation and SNP genotyping analysis

Tissue processing and DNA isolation were carried out as described previously [49]. Genotyping of the SNPs was performed using TaqMan SNP™ Genotyping Assays (rs580041: C_778399_20; SNP rs922224: C_8945633_10; SNP rs7587026: C_3041377_10; Applied Biosystems, Foster City, CA, USA) according to the manufacturer’s protocol on an ABI PRISM 9700HT sequence detection system (PE Applied Biosystems) [44]. The assignments of the alleles of the SNPs refer to the forward genome strand orientation relative to the NCBI reference genome build 36. Allelic discrimination was carried out using the SDS 2.2 software.

#### RNA isolation and mRNA expression analysis

Total RNA for gene expression microarray analysis was isolated from human hippocampal tissue samples using All Prep DNA/RNA Mini Kit (Qiagen) according to the manufacturer’s protocol. To synthesize cDNA from total RNA and in vitro transcription to biotin-labelled cRNA, Illumina Total Prep-96 RNA Amplification Kit (Life Technologies Corporation, Darmstadt) was used according to manufacturer’s protocol. cRNA was then hybridized on Human HT-12v3 Expression Bead Chips using lllumina Direct Hybridization Assay Kit (Life Technologies Corporation, Darmstadt, Germany). The Illumina Bead Array Reader was applied for scanning and the data were analysed using Illumina’s Genome Studio Gene Expression Module. Gene expression data were normalised using the Illumina Bead Studio software suite by means of quantile normalisation with background subtraction.

#### Analysis of severity of neuronal cell loss in different hippocampal subfields

To assess the possibility that the observed effect of rs7587026 on *SCN1A* expression might be compounded by differences in severity of hippocampal sclerosis (HS), we used immunohistochemistry for NeuN (encoded by *RBFOX3*) to quantify neuronal loss. Among the 91 individuals included in the *SCN1A* expression analysis, suitable samples for immunohistochemistry were available for 73. Neuronal loss was categorised qualitatively as severe, moderate, mild, or no neuronal loss by experienced neuropathologists as described previously [4].

#### Statistical analyses

Statistical analyses were performed using GraphPad Prism 9.00 (San Diego, CA, USA). Differences in *SCN1A* expression and neuronal loss by genotype were assessed using a Kruskal–Wallis test with a post hoc Dunn’s test. The significance level was set at *P* < 0.05.

### Patterns of neuronal loss in surgical hippocampal samples from individuals with MTLEHS—replication

To replicate and quantify the analysis of neuronal loss, we employed postsurgical samples from therapeutic anterior temporal lobectomies from individuals with MTLEHS archived in the UCL Epilepsy Society Brain and Tissue Bank. The maximum available number of cases with sufficient tissue for diagnosing HS as per ILAE guidelines [5] and accessible genetic data on rs7587026 were chosen (96 in total). The study has ethical approval (UK National Research Ethics 17/SC/0573) and informed consent was obtained from all participants.

For visualisation of the extent of neuronal loss, two sections were immunostained per case, one with NeuN, and the other with MAP2. We compared the total percentage area labelled for each region of interest (ROI)—CA1, CA2, CA3, CA4—across the three genotypes, separately for NeuN and MAP2-stained slides.

#### Immunohistochemistry

A representative formalin-fixed and paraffin-embedded tissue block was selected from the hippocampal resection which showed maximal representation of all subfields. Immunohistochemistry for NeuN and MAP2 was performed on 5 µm thick tissue sections on the Bond-MAX Autostainer (Leica, UK). The NeuN antibody was mouse clone A60 (Chemicon/Millipore, UK, diluted 1:2000). The MAP2 antibody was mouse clone HM-2 (Sigma-Aldrich, UK, diluted 1:5000). The sections were dewaxed and rehydrated, and put through heat-induced antigen retrieval (citrate buffer pH 5.9–6.1, Epitope Retrieval Solution 1), at 100 °C for either 20 min (NeuN) or 30 min (MAP2). Hydrogen peroxide block was carried out for 20 min, followed by primary antibody incubation for 15 min. Slides were incubated first with the blocking agent, post-primary horseradish peroxidase (HRP; Bond Polymer Refine Red Detection, Leica, UK), for 8 min, and then in polymer HRP (Bond Polymer Refine Red Detection, Leica, UK) for further 8 min. For both markers, immunocomplexes were then visualized with 3,3’-Diaminobenzidine (DAB), followed by 5 min incubation with DAB enhancer. Slides were counterstained with haematoxylin, dehydrated and coverslipped. Between each step, all sections were washed with phosphate-buffered saline (Fisher Scientific, Ltd., UK). Negative controls were run simultaneously but without primary antibodies. Slides were digitized using a whole slide scanner (Leica SCN400 scanner, Leica, UK) at × 40 magnification. Images were acquired using Leica SlidePath Digital Image Hub software (Leica, UK).

#### Quantitative analysis

On each digitised slide, four ROIs were manually selected—CA1, CA2, CA3, and CA4. The percentage of light brown and dark brown staining within each selected region was then calculated by the Definiens Developer XD 64 Life software (Definiens AG Munich, Germany), giving the total percentage of staining for each ROI, with higher values implying greater preservation of the respective region. For a subset of 10 samples, the assessment was carried out by two separate researchers, with excellent internal consistency (Cronbach alpha coefficient *α* = 0.994 for NeuN and *α* = 0.985 for MAP2).

#### Statistical analysis

Statistical analysis was carried out with SPSS (versions 24 for Windows and 25 for Mac, IBM Corp, Armonk, NY). GraphPad Prism 9.00 (San Diego, CA, USA) was used for the graphic representation of the data. In case of any missing values, the individual was excluded for the analysis of that ROI. Data were not normally distributed and Mann–Whitney *U* tests were used to compare staining between samples from individuals with rs7587026 genotypes AA and AC, and genotypes AA and CC. A two-tailed value of *P* ≤ 0.05 was considered significant.

### Hippocampal and amygdalar MRI volume measurements in healthy individuals by rs7587026 type

To determine whether rs7587026 minor allele homozygosity influences brain anatomy, we examined, using high-resolution structural brain MR imaging data, the volume of subcortical structures typically associated with MTLEHS (hippocampus, amygdala, and thalamus), and total intracranial volume (ICV) in 597 healthy individuals with available genotype data. All eligible participants were included from the Queensland Twin Imaging (QTIM) study, a longitudinal study of healthy young twins and their siblings who underwent neuroimaging, genetic, and comprehensive cognitive assessments [66]. The QTIM study was approved by the Human Research Ethics Committees of the University of Queensland and the QIMR Berghofer Medical Research Institute. Informed consent was obtained from each participant. See Supplementary Methods (online resource) for full inclusion criteria. For the present analysis, one member of each set of monozygotic twins was randomly excluded.

#### Genotyping

Genome-wide genotype data were collected on the Human610-Quad BeadChip (Illumina, Inc., San Diego, CA), and subjected to standard quality control measures used in large genome-wide association study (GWAS) analyses. Genotype for rs7587026 was extracted for each individual.

#### Brain MRI acquisition and processing

High-resolution structural MRI scans were obtained on a single 4-Tesla scanner (Bruker Medspec) using the same imaging protocol. Three-dimensional T1-weighted images were acquired with an inversion recovery rapid gradient echo sequence (TI/TR/TE = 700/1500/3.35 ms; flip angle = 8°; slice thickness = 0.9 mm). MR Images were processed using FreeSurfer (http://surf.nmr.mgh.harvard.edu/) [11, 16–18]. Quality control of image segmentation was performed according to the Enhancing Neuro Imaging Genetics through Meta-Analysis (ENIGMA) Consortium procedures (http://enigma.ini.usc.edu/protocols/imaging-protocols) [52].

#### Data analysis

Differences in mean hippocampal, amygdalar and other subcortical (thalamus, caudate nucleus, putamen, and globus pallidus) volume measures by genotype were examined using generalized linear regression models (covariates: ICV, age, gender, and zygosity). The mean was calculated from left and right subcortical structural volumes in mm^3^. Differences in participants’ age by rs7587026 genotype were examined using an unpaired, two-tailed *t* test. Sex and zygosity differences between groups were tested using a chi-squared test. An alpha-level of 0.05 was used to determine statistical significance. All statistical analyses were performed using the R statistics package (https://www.r-project.org) [55].

### Generation of *scn1lab*-overexpressing zebrafish larvae and experimental setup

To study the functional consequences of increased *SCN1A* expression, we generated, using transposon-mediated bacterial artificial chromosome (BAC) transgenesis, a zebrafish line expressing exogenous *scn1a* (in zebrafish known as *scn1lab*) (*scn1lab*-OE). We studied the seizure propensity of the *scn1lab*-OE larvae using EEG, exposure to sodium channel blocking antiseizure medications, and exposure to hyperthermia.

#### Ethical approval

All experiments were performed in compliance with the European Community Council Directive of November 2010 for Care and Use of Laboratory Animals (Directive 2010/63/EU), and the ARRIVE guidelines. The Norwegian Food Safety Authority via its experimental animal administration’s supervisory and application system approved all animal experimentation (FOTS ID 15469 and 23935).

#### Construct generation and zebrafish husbandry

The CH211-74H7 clone in a pTARBAC2.1 vector (Source BioScience CHORB736H0774Q) was modified to contain Tol2 elements flanking either side of *scn1lab*. For the over-expression construct, an mCherry reporter was linked to the last exon of *scn1lab* with a self-cleaving T2A sequence to separate the fluorescence reporter and scn1a proteins after translation. As a control construct, mCherry was placed upstream of the first exon of *scn1lab*, preventing transcription of *scn1lab*. BAC constructs (20 ng/μl) were injected together with capped tol2 transposase mRNA (50 ng/μl) into the cytoplasm of 1-cell stage fertilized embryos, at a volume of 1.5 nl.

Adult zebrafish of the AB strain were raised under standardized aquaculture conditions, in a 14/10 h light/dark cycle at 28.5 °C. Eggs from natural spawning of adult fish were collected and microinjected with the BAC construct. Next, injected eggs were transferred to petri dishes and raised until 4 day post-fertilization (dpf) in embryo medium (17 mM NaCl, 2 mM KCl, 1.8 mM Ca(NO_3_)_2_, 0.12 mM MgSO_4_, 1.5 mM HEPES buffer pH 7.1–7.3 and 0.6 μM methylene blue; 14/10 h dark/light cycle at 28.5 °C). 3 or 4 dpf larvae were screened using fluorescence stereomicroscopy. Only those larvae which expressed the BAC fluorescent reporter in the brain were used for experiments.

#### Morphological assessment

4 dpf larvae were photographed using a Leica M205 FA stereomicroscope and assembled using Adobe Photoshop 2020. All pictures were taken at the same resolution.

#### EEG recordings

We performed EEG analysis on larval optic tecta as described by Afrikanova et al. [2]. Epileptiform-like discharges were detected by inserting a glass electrode filled with artificial cerebrospinal fluid (124 mM NaCl, 2 mM KCl, 2 mM MgSO_4_, 2 Mm CaCl_2_, 1.25 mM KH_2_PO_4_, 26 mM NaHCO_3_, 10 mM glucose) into the optic tectum of individual 4 dpf zebrafish larvae for 20 min (MultiClamp 700B amplifier, Digidata 1550 digitizer, Axon instruments, USA). The larvae were restrained with the aid of a thin layer of 2% low melting point agarose. The Clampfit version 10.6.2 software (Molecular Devices Corporation, USA) was used for processing the EEG recordings. The data were analysed manually by a highly trained observer, blind to treatment group.

#### Drugs

We tested the response of 3 dpf *scn1lab-*OE larvae to three different sodium channels blockers, namely, phenytoin (100 µM; Sigma Aldrich), oxcarbazepine (170 µM; Sigma Aldrich) and valproic acid (100 µM; Sanofi Aventis). All drugs were dissolved in dimethyl sulfoxide (DMSO) at a final concentration of 0.5% v/v DMSO (Sigma Aldrich). The appropriate vehicle control was prepared by dissolving DMSO in zebrafish medium. 3-day-old larvae were screened under fluorescence microscope, and subsequently treated for 20 h in respective drugs. Next, EEG recordings were conducted as detailed above.

#### qRT-PCR

To determine whether the level of *scn1lab* overexpression correlates with the number of seizures in *scn1lab-*OE, following EEG recording, each single larva was collected for qRT-PCR analysis. Larvae were transferred to Eppendorf tubes filled with 200 µl Trizol. mRNA was purified from single larvae as described by Dupret et al.[12] Next, cDNA was synthesized using SuperScript™ IV First-Strand Synthesis System (Invitrogen) and amplified using PowerUp™ SYBR™ Green Master Mix (Applied Biosystems) according to manufacturer’s instructions. Relative enrichment was computed according to the 2^−∆∆t^ method [38]. Expression levels were normalized against glyceraldehyde 3-phosphate dehydrogenase (*gapdh),* which at the stage of larval development used in this study (i.e., 4 dpf), has been shown to be stably expressed [6, 42].

#### Hyperthermia-induced abnormalities

To explore possible effects of a rapid ambient temperature increase on seizure propensity in relation to *scn1lab* overexpression, 4-day-old *scn1lab-*OE or control larvae were transferred to 50 ml falcon tubes filled with 10 ml of pre-heated (33, 35 or 37 °C) medium. Next, tubes were bathed in water bath (33, 35 or 37 °C) for 5 min. Subsequently, larvae were monitored for 5 min under a microscope for occurrence of convulsion-like behavior, including repetitive pectoral fin fluttering, lying on one side (loss of posture), tail wagging, or myoclonus-like jerks by a highly experienced researcher, blinded to the group of animals being scored. Larvae were scored for heat-induced convulsive-like phenotype.

#### Statistical analysis

For statistical purposes, GraphPad Prism 8.00 (San Diego, CA, USA) was used. For comparisons, one-way or two-way ANOVA with Tukey’s or Sidak’s multiple comparisons test was used as appropriate. All data are shown as mean ± standard error of the mean (SEM).

### Bioinformatics

We examined the genomic sequence context of rs7587026 using GENCODE [20] release v28. As the GENCODE project is incomplete, we also reviewed transcriptomics data not yet incorporated into the annotation catalogue. These data sets included RNAseq read coverage graphs (from HPA [28, 60]), RNAseq model collections (including FANTOM [24], GTEx [39] and PLAR [23]) and long-read RNA libraries (from GENCODE [34, 58]). We then employed the ReMap resource [8], which seeks to characterise regulatory elements via the large-scale curation and integration of publicly available ChIP-seq data sets (see Supplementary Material in online resource for more details).

#### Human sequence constraint

The map of sequence constraint for the human genome created by di Iulio et al. in 2018 was used to identify sequences that are rarely mutated in healthy individuals, intolerant to genetic variation and thus more likely to be functionally relevant [27]. The map, which was produced using whole-genome sequencing (WGS) data from 11,257 individuals, assigns a context-dependent tolerance score (CDTS) to each 10 bp long bin of the genome, indicating the likelihood of variation: the lower the score, the less frequently the bin is affected by variation, and the more mutation intolerant the bin is [27].

#### Linkage disequilibrium (LD) blocks

The BigLD function from the gpart R package was used to detect the LD block structure of the *SCN1A* locus. Big-LD is a block-partitioning algorithm that estimates the LD block organisation of the genetic region of interest using the interval graph modelling of LD bins (clusters of strong pairwise LD SNPs) [31]. The BigLD algorithm employs an agglomerative strategy, which involves detecting small communities of SNPs in strong LD and then merging them if they share SNPs in high LD, ultimately resulting in larger LD blocks than other alternative methods. To account for this feature, the LD block structure of the *SCN1A* locus was also estimated using the command line tool PLINK 1.9 [7, 46].

#### Transcription start sites (TSSs)

The functional annotation of the mammalian genome (FANTOM) 5 data repository was used. The FANTOM5 project performed cap analysis gene expression (CAGE) across 975 samples, including human primary cells, tissue samples and cancer cell lines and mapped transcription start sites (TSSs) throughout the genome and their differential usage [1, 19]. Using the TSS peaks identified by the FANTOM5 project, the genetic location of TSSs in the *SCN1A* locus was explored. The FANTOM5 project also measured the relative activity of each TSS as normalized tags per million (TPM), calculated using the relative log expression (RLE) method in edgeR [1, 19, 47]. The relative activity of the TSSs in the *SCN1A* locus was compared across hippocampus and cortex samples.

### In vitro regulatory analysis

To investigate whether the *SCN1A* region comprising the SNP rs7587026 area possesses regulatory activity can confer promoter activity and, if so, whether this promoter activity is differentially affected by genotype, we cloned a 50 bp fragment surrounding the SNP into a luciferase reporter vector. This fragment was chosen as according to the UCSC Genome Browser, it has a high conservation score among 100 vertebrates [30]. In our experimental experience, this feature in particular provides a strong argument for biologically relevant genomic motifs [40, 63]. The conservation score broke down markedly up- and downstream of this 50 bp fragment [30]. Furthermore, two large repeats flanking the SNP region prevented extension of the luciferase fragment: L1M4, a long interspersed nuclear element (LINE) located at 300 bp from the SNP on the 5’ side, and ERVL–MaLR, a long terminal repeat (LTR) located at 90 bp from the SNP on the 3’.

The fragment was cloned in the sense direction respective to the ATG-start codon; therefore, in this section, the major allele or wild type allele (C) is denoted as G and the minor allele (A) as T. Luciferase constructs are denoted as *SCN1A*-50 bp-rs7587026-G(WT)-Luciferase and *SCN1A*-50 bp-rs7587026-T-Luciferase. Bioinformatic analyses had revealed a consensus binding motif for the transcription factor Sox2 which contained the SNP. Therefore, we generated two additional luciferase reporter constructs, one that comprised only the Sox2 binding site for each genotype (*SCN1A*-20 bp-rs7587026-G(WT)-Luciferase and *SCN1A*-20 bp-rs7587026-T-Luciferase) and one in which the Sox2 binding site had been replaced by a scrambled sequence.

#### Ethical approval

Approval was obtained from the Ethical Commission of University Hospital Bonn (196/17).

#### Cloning

Luciferase constructs containing a 50 bp fragment surrounding rs7587026 were generated by in-fusion cloning. Fragments were PCR amplified from human genomic DNA isolated from blood either homozygous for the major (wild type) allele G or the minor allele T (see primers in Supplementary Table 1, online resource). The PCR product was cloned in the NheI/BglII sites of the pGL3-basic vector (Promega) generating the constructs *SCN1A*-50 bp-rs7587026-G(WT)-Luciferase and *SCN1A*-50 bp-rs7587026-T-Luciferase. To generate the constructs *SCN1A*-50 bp-Scramble3-Luciferase, *SCN1A*-20 bp-rs7587026-G(WT)-Luciferase and *SCN1A*-20 bp-rs7587026-T-Luciferase oligonucleotides (see oligonucleotide sequence in Supplementary Table 1, online resource) were first annealed and inserted in the NheI/BglII sites of the pGL3-basic vector (Promega).

#### Cell culture, transfection and luciferase assay

NS20Y cells (Sigma, 08062517) were cultured in DMEM (Sigma, D6546) supplemented with 10% (v/v) heat inactivated FBS, 2 mM L-Glutamine, 100 units/ml penicillin/streptomycin and kept at 37 °C and 5% CO2. Cells were plated in 24-well plates and after 24 h transfected using Lipofectamine (Invitrogen) following the manufacturer ´s instructions. Transfection was performed using 100 ng of the luciferase constructs and 25 ng of the Renilla luciferase control construct (Promega) and collected 48 h after transfection. Luciferase assay were performed as described before [59].

HEK293T cells were cultured in DMEM (Invitrogen) supplemented with 10% FCS and 1% penicillin–streptomycin and kept at 37 °C and 5% CO2. Cells were plated in 10 cm2 culture dishes and transfected using calcium phosphate with 10 µg of the constructs: pCMV-Sox2-T2A-GFP (Addgene plasmid #127537) or hypB-CAG-2A-eGFP (derived from a PiggyBac backbone) used as a control. Proteins were isolated 48 h after transfection.

#### Protein extraction and electrophoretic mobility shift assay (EMSA)

Nuclear protein extracts prepared either from mouse brain or from HEK293T cells transfected with pCMV-Sox2-T2A-GFP (Addgene#127537) was performed using the NE-PER kit (Thermo Fisher Scientific, #78833) following the manufacturer´s instruction. Adult male mice (∼50 days,  > 20 g) were obtained from Charles River (C57Bl/6-N). Animals were decapitated under deep isoflurane (Forene) anaesthesia. Brains were prepared, the cerebellum was removed and the right hemisphere used for nuclear protein isolation.

The oligonucleotides for EMSA (Supplementary Table 2, online resource) were annealed in annealing buffer (10 mM Tris pH 7.5, 50 mM NaCl, 1 mM EDTA) by incubation for 2 min at 95 °C followed by 1 h of slowly cooling down. The EMSA reaction was performed using the commercially available EMSA kit (Invitrogen, #E33075) following the manufacturer’s instructions. Briefly, 0.5 µg of annealed oligonucleotides and 5 µg of nuclear protein extract were mixed in 1X binding buffer and incubated at 30 °C for 30 min. Following incubation, samples were mixed with 6X EMSA loading solution. The reaction was separated in 6% nondenaturing polyacrylamide gels. Gels were stained with SYBR Green for 20 min and imaged.

#### Statistical analysis

Data for luciferase activity are shown as mean ± standard error of the mean (SEM). Activity in different conditions was compared using one-way ANOVA, with Tukey’s test for multiple comparisons, or one-sample *t* test as appropriate.

#### Data availability

The data that support the findings of this study are available from the corresponding author upon reasonable request and subject to local requirements for each source data set.

## Results

### *SCN1A* mRNA expression levels in hippocampi of patients with pharmacoresistant MTLEHS differ by genotype

**Fig. 1:**
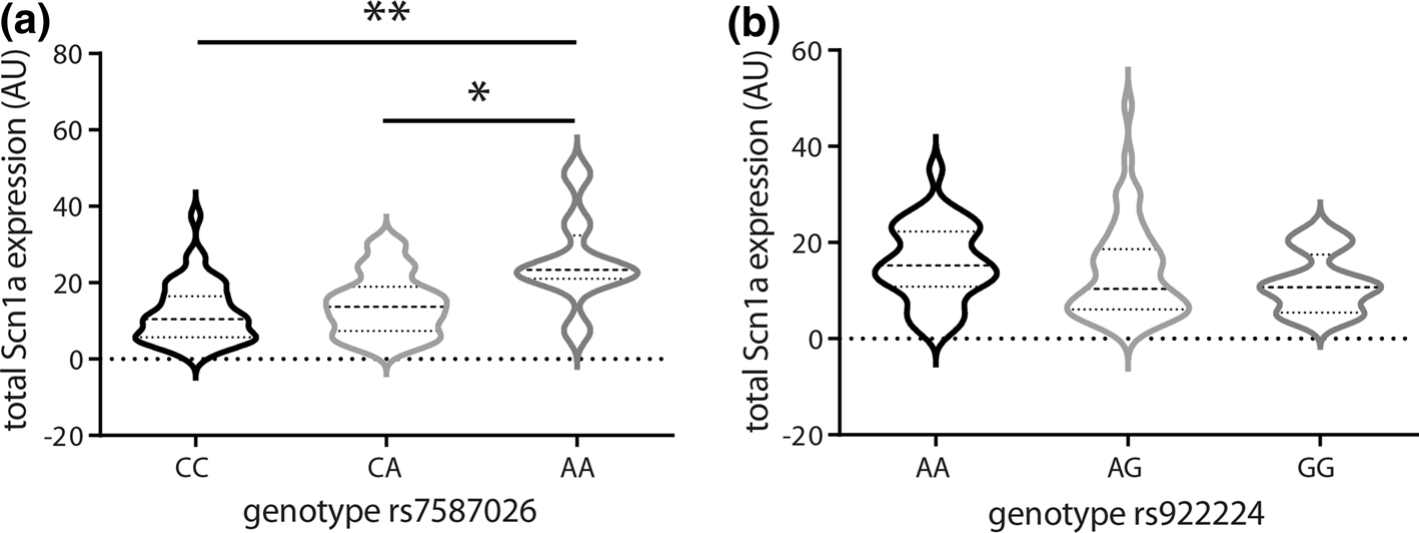
rs7587026 genotype is correlated with *SCN1A* expression in individuals with MTLEHS, whereas rs922224 genotype is not. **a**,**b** Violin plots showing the expression levels of *SCN1A* in hippocampi of individuals with MTLEHS stratified by SNP genotype. Horizontal lines within the plots present median and quartiles. **a** Individuals with MTLEHS homozygous for the minor allele (rs7587026) have higher *SCN1A* expression levels compared to the other two genotypes (CC: *n* = 43, CA: *n* = 40, AA: *n* = 8; Kruskal–Wallis test: **P = 0.004, Dunn’s test: CC vs. AA: **P = 0.003, CA vs. AA: *P = 0.035). **b** No differences in *SCN1A* expression levels were observed by rs922224 genotype in MTLEHS (AA: *n* = 33, AG: *n* = 46, GG: *n* = 12; Kruskal–Wallis test: *P* = 0.226). See Supplementary Table 3 (online resource) for the expression level values

Hippocampal *SCN1A* expression differed by rs7587026 genotype (Kruskal–Wallis test *H* = 10.92, *P* = 0.004). Individuals homozygous for the minor allele (AA; *n* = 8) showed significantly increased expression compared to those homozygous for the major allele (CC; *n* = 43; Dunn’s test: *P* = 0.003; Fig. 1a), and to heterozygotes (AC; *n* = 40; Dunn’s test: *P* = 0.035). Neither rs922224 nor rs6432860 genotypes showed association with hippocampal *SCN1A* expression (Kruskal–Wallis test for rs922224: *H* = 2.98, *P* = 0.226; Fig. 1b; for rs6432860, see Supplementary Fig. 1, online resource).

### rs7587026 type is not associated with severity of hippocampal cell loss in MTLEHS

No significant correlation was identified between allelic variants and degree of hippocampal cell loss in any analysed subregions (CA1–CA4; Supplementary Fig. 2, online resource) for the samples in which expression was studied (detailed in 1 above).

We examined this question in a second independent cohort also. The cases comprised nine rs7587026 minor allele homozygotes (AA), 35 heterozygotes (CA), and 52 major allele homozygotes (CC). For NeuN, median percentage staining ranged from 0.49 (AC in CA4) to 4.36 (AA in CA2). For MAP2, median percentage staining ranged from 31.1 (AA in CA1) to 87.2 (AA in CA2). No statistically significant differences in degree of staining by genotype were observed for any of the ROIs using either immunolabel (Supplementary Fig. 3 and Supplementary Tables 4 and 5, online resource).

### Hippocampal and amygdalar volumes differ by rs7587026 genotype as measured by MRI in healthy individuals

**Fig. 2:**
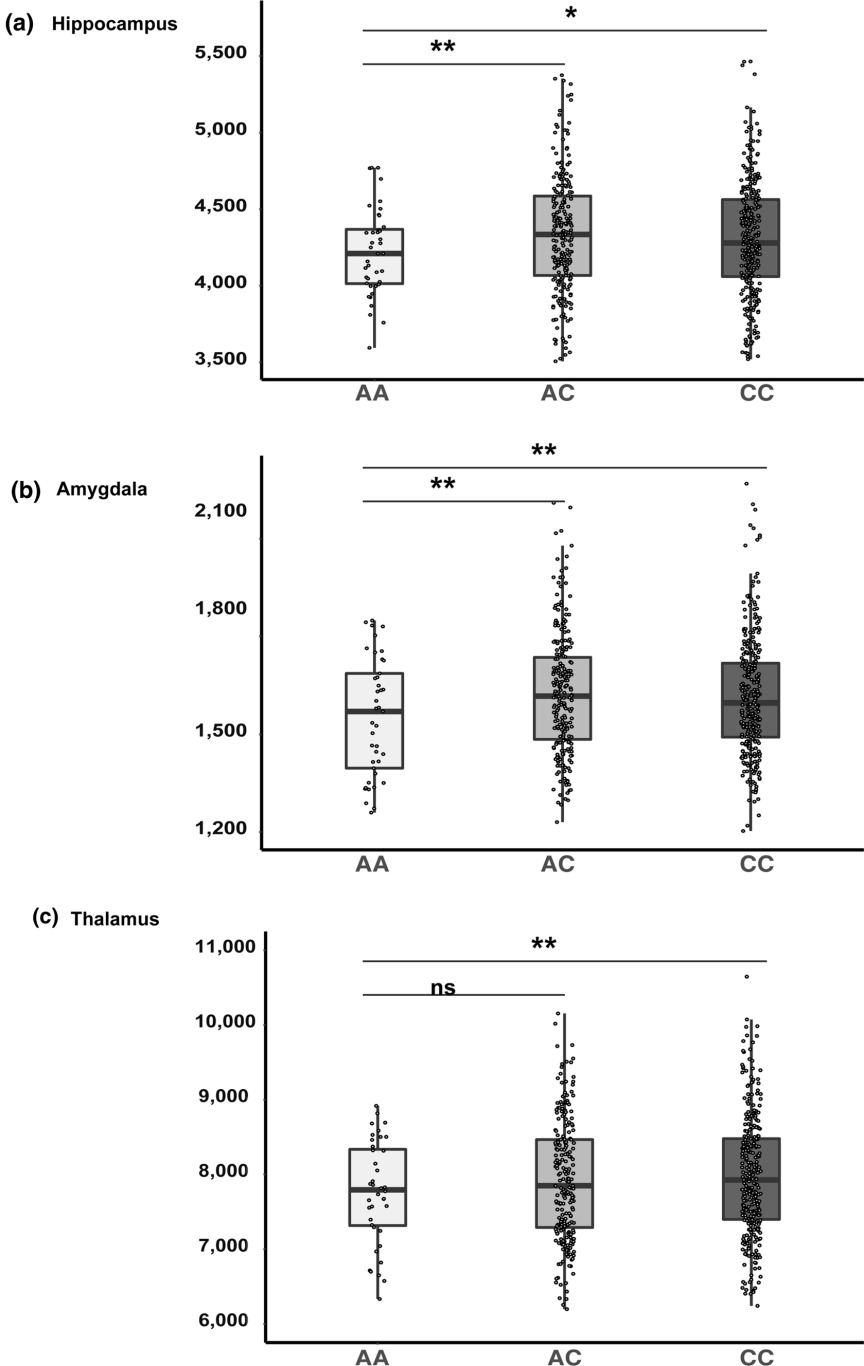
Subcortical volumes by genotype. The mean volumes of the **a** hippocampus, **b** amygdala and **c** thalamus are presented based on rs7587026 genotype in healthy young individuals. The mean volume was calculated from left and right hemisphere structural volumes in mm^3^. In each figure, the x-axis displays three groups of the QTIM sample based on rs7587026 genotype: minor allele homozygotes (AA; *n* = 41), minor allele heterozygotes (AC; *n* = 242), and major allele homozygotes (CC; *n* = 314). The y-axis displays the structural volume in mm^3^. Error bars represent standard error (SE) of the means. *P < 0.05; **P < 0.01; *ns* non-significant

Among the 597 individuals, 236 were male and 361 female, with a mean age of 23.5 years (standard deviation ± 3.1; Supplementary Table 6, online resource). Hippocampal, amygdalar, and thalamic volumes are presented in Fig. 2 and Table 1. Minor allele homozygotes (AA; *n* = 41) displayed significantly reduced mean hippocampal volume compared to major allele homozygotes (CC; *n* = 314; Cohen’s *D* = − 0.28, *P* = 0.02), and to heterozygotes (AC; *n* = 242, Cohen’s *D* = − 0.36, *P* = 0.009; Fig. 2a). AA homozygotes also displayed reduced mean amygdalar volume relative to CC homozygotes (Cohen’s *D* = − 0.35, *P* = 0.01) and heterozygotes (Cohen’s *D* = − 0.39, *P* = 0.004) (Fig. 2b). Similarly, AA homozygotes showed significantly reduced mean thalamic volume relative to CC homozygotes (Cohen’s *D* = − 0.29, *P* = 0.009) and a trend of reduced thalamic volume when compared to heterozygotes (Cohen’s *D* = − 0.21, *P* = 0.07; Fig. 2c). Other subcortical volumes, including the caudate nucleus, putamen, and globus pallidus, did not differ by rs7587026 genotype (Table 1).

**Table 1:** Mean volumes of subcortical structures in healthy participants according to rs7587026 type

	AA Mean (SD)	AC Mean (SD)	CC Mean (SD)	AA vs. AC		AA vs. CC	
				Cohen’s D	*P* value	Cohen’s D	*P* value
Hippocampus	4198 (308)	4333 (389)	4307 (402)	− 0.36	0.009	− 0.28	0.02
Amygdala	1552 (180)	1626 (186)	1616 (187)	− 0.39	0.004	− 0.35	0.01
Thalamus	7756 (683)	7925 (805)	7985 (834)	− 0.21	0.07	− 0.29	0.009
Caudate nucleus	3790 (499)	3866 (505)	3915 (494)	− 0.15	0.24	− 0.25	0.07
Putamen	5695 (629)	5716 (663)	5740 (684)	− 0.03	0.78	− 0.07	0.60
Globus Pallidum	1399 (174)	1433 (192)	1445 (203)	− 0.17	0.22	− 0.22	0.09

Volumes are expressed in mm^3^

### *scn1a-*overexpressing zebrafish larvae exhibit spontaneous seizures and are more prone to heat-induced convulsions

**Fig. 3:**
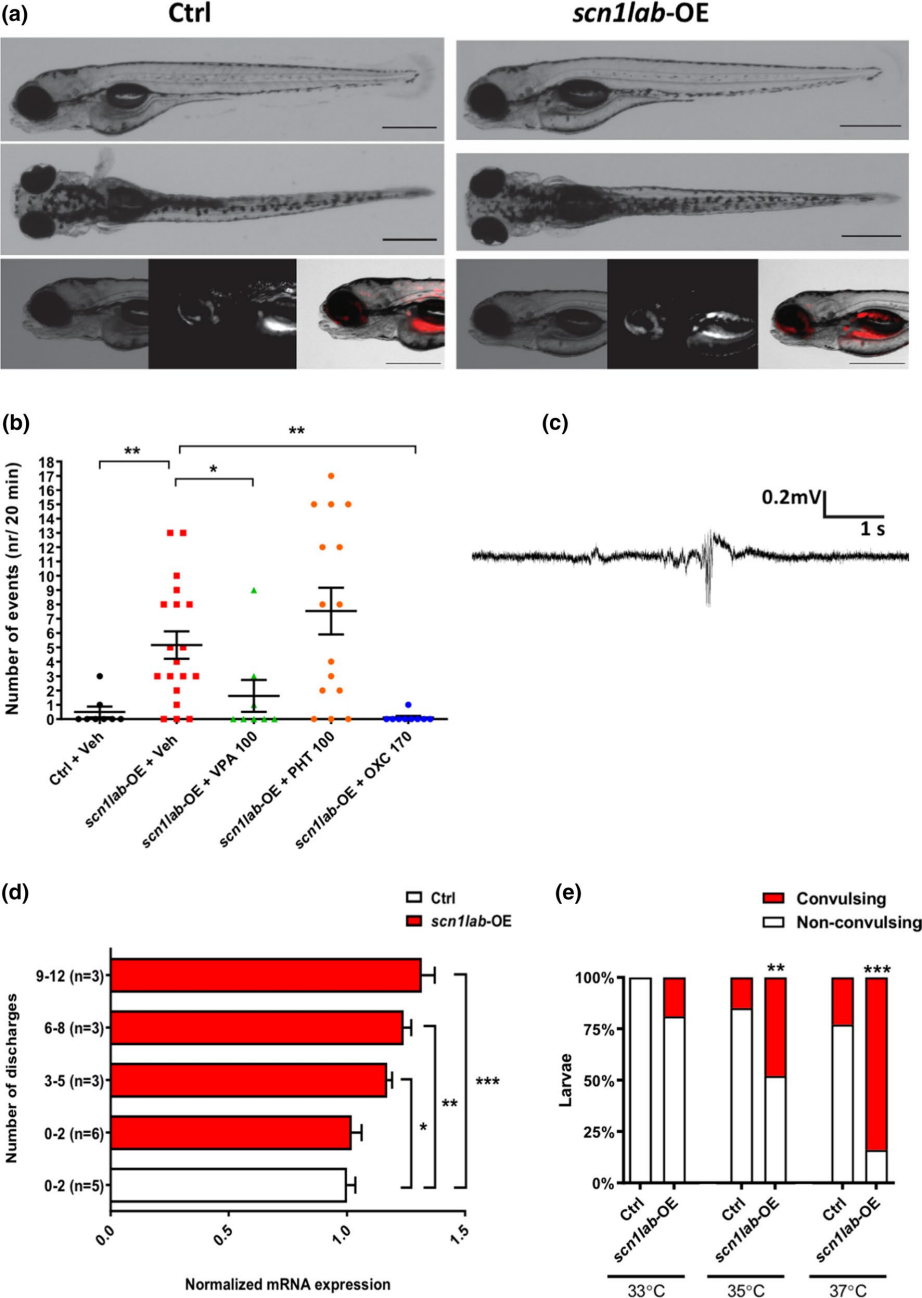
Overexpression of *scn1a* in zebrafish increases seizure susceptibility and temperature sensitivity. **a** Representative images of control (Ctrl; left panel) and *scn1lab*-OE larvae (right panel). Red colour depicts mCherry fluorescence. **b** Number of EEG discharges in control and *scn1lab*-OE larvae. Horizontal bars represent mean ± SEM. Sample sizes: Ctrl, *n* = 8; *scn1lab*-OE + Veh, *n* = 19; *scn1lab*-OE + valproic acid (VPA) (100 µM), *n* = 8; *scn1lab*-OE + phenytoin (PHT) (100 µM), *n* = 15; *scn1lab*-OE + oxcarbazepine (OXC) (170 µM), *n* = 9. **P < 0.01, *P < 0.05 (One-way ANOVA with Tukey’s *post-hoc*). Abbreviations: VPA-valproic acid, PHT-phenytoin, OXC- oxcarbazepine. **c** An example of EEG recordings obtained from the optic tecta of 4 dpf *scn1lab*-OE larvae. **d** Number of discharges recorded in *scn1lab*-OE larvae relative to scn1lab mRNA levels. Vertical bars represent mean ± SEM. **e** Percentage of larvae developing heat-induced seizures. Sample sizes: Ctrl, *n* = 18, *n* = 20 or *n* = 22 (for 33, 35 or 37 °C, respectively); *scn1lab*-OE, *n* = 16, *n* = 19 or *n* = 18 (for 33, 35 or 37 °C, respectively). ***P < 0.001, **P < 0.01 (Two-way ANOVA with Sidak’s multiple comparisons test)

Morphologically, *scn1lab*-overexpressing (hereafter, *scn1lab-*OE) larvae did not exhibit any overt malformations (Fig. 3a), though a subset were slightly hyperpigmented and occasionally lacked a swim bladder. Most (16/19) *scn1lab-*OE larvae exhibited spontaneous seizures in the form of high-voltage spikes, spike-wave complexes and polyspike-wave discharges, while only 2/8 control larvae displayed only a single seizure [one-way ANOVA *F*(4,54) = 6.95 (*P* < 0.001); Fig. 3b, c]. As predicted, oxcarbazepine (*P* < 0.01) and valproic acid (*P* < 0.05) led to a decreased number of EEG discharges in *scn1lab-*OE larvae. Interestingly, phenytoin did not exert antiseizure activity in *scn1lab-*OE larvae (Fig. 3b).

The number of EEG discharges in *scn1lab-*OE larvae differed by the level of *scn1lab* overexpression [one-way ANOVA *F*(4,15) = 10.75 (*P* < 0.001); Fig. 3d] and was remarkably dose-sensitive. Even a modest rise in *scn1lab* transcript levels of 11% was sufficient to increase seizures to between 3 and 5 per 20 min recording (*P* < 0.05), while 24% and 31% increases resulted in 6 to 8 (*P* < 0.01) and 9 to 12 seizures (*P* < 0.001) in *scn1lab-*OE larvae, respectively.

To explore possible effects of a rapid ambient temperature increase on seizure propensity in relation to *scn1lab* overexpression, we bathed control and *scn1lab-*OE larvae (normally reared at 28.5 °C) in embryo medium pre-heated to 33, 35 or 37 °C for 5 min. We then assessed larvae for convulsion-like behavior (i.e., loss of posture, excess tail beating, pectoral fin fluttering). Here, two-way ANOVA with Sidak’s multiple comparisons test revealed a difference between tested groups of animals [group of animals *F*(1,6) = 88.33, *P* < 0.001; temperature *F*(2,6) = 39.41, *P* < 0.001; group of animals × temperature interaction *F*(2,6) = 8.87, *P* < 0.05; Fig. 3e]. Statistically significant differences in the number of fish showing convulsive-like behaviors were observed between control and *scn1lab-*OE larvae on exposure to 35 °C (*P* < 0.01) and 37 °C (*P* < 0.001), but not 33 °C (Fig. 3e).

### rs7587026: bioinformatic analysis

rs7587026 does not overlap with any exonic sequences found in GENCODE [20] release v28, being instead found within intronic sequence of the *SCN1A* 5' untranslated region (as well as intronic sequence of lncRNA ENSG00000236107 on the opposite strand). Our extensive investigations of other data sets including RNAseq read coverage graphs (from HPA [28, 60]), RNAseq model collections (including FANTOM, [24] GTEx [39] and PLAR [23]) and long-read RNA libraries (from GENCODE [34, 58]) did not did not suggest that rs7587026 is exonic. Using the ReMap resource [8], in silico analysis showed that the variant falls within DNA binding regions of the transcription factor SOX2 but also of other molecules including AR proteins (see also Supplementary Material in online resource).

**Fig. 4:**
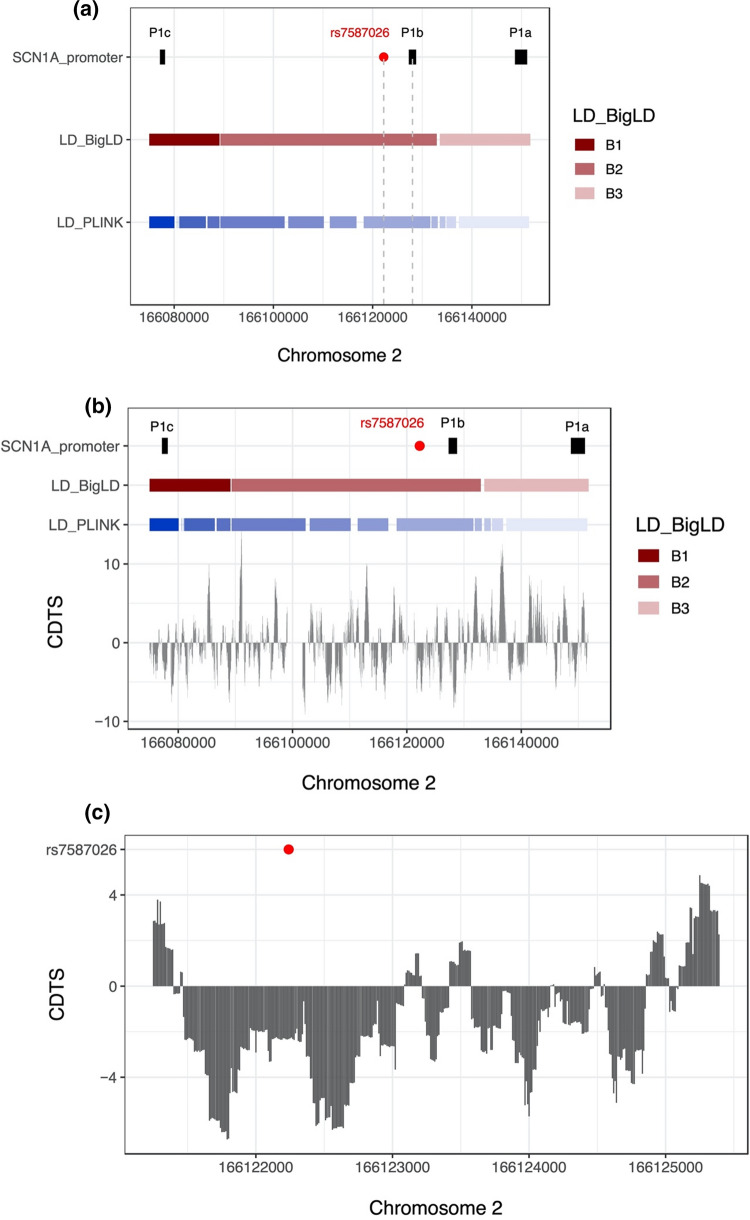
**a** Chromosomal position of rs7587026 with respect to the three known *SCN1A* promoter regions. rs7587026 falls within the same LD block as the P1b promoter. X-axis: position on chromosome 2, Y-axis: elements that were considered (*SCN1A* promoter regions, LD blocks defined using the BigLD function and PLINK). **b** The top half of the figure shows the genetic position of the three *SCN1A* promoter regions, rs7587026, as well as a representation of the LD blocks that span the locus; the bottom half shows the sequence constraint level. **c** Level of sequence constraint of the genetic region surrounding rs7587026. Sequence constraint is expressed as context-dependent tolerance score (CDTS), indicating the likelihood of variation. Negative CDTS scores indicate highly constrained regions, infrequently mutated in healthy individuals and more likely to be functionally relevant

According to both the BigLD and PLINK estimates of LD blocks, rs7587026 is located between the P1b and P1c *SCN1A* promoters, in the same LD block as P1b (Fig. 4). In terms of the level of sequence constraint, rs7587026 falls within a broader genetic region characterised by a negative CDTS score, indicating a highly constrained region of the human genome, that is infrequently mutated in healthy individuals (Fig. 4). In the di Iulio et al. map [27], the CDTS score for the 10 bp bin upstream of rs7587026 (chr2:166,122,230–166,122,240) was -2.3445, and the score for the 10 bp bin downstream of rs7587026 (chr2:166,122,240–166,122,250) was − 2.3232.

**Fig. 5:**
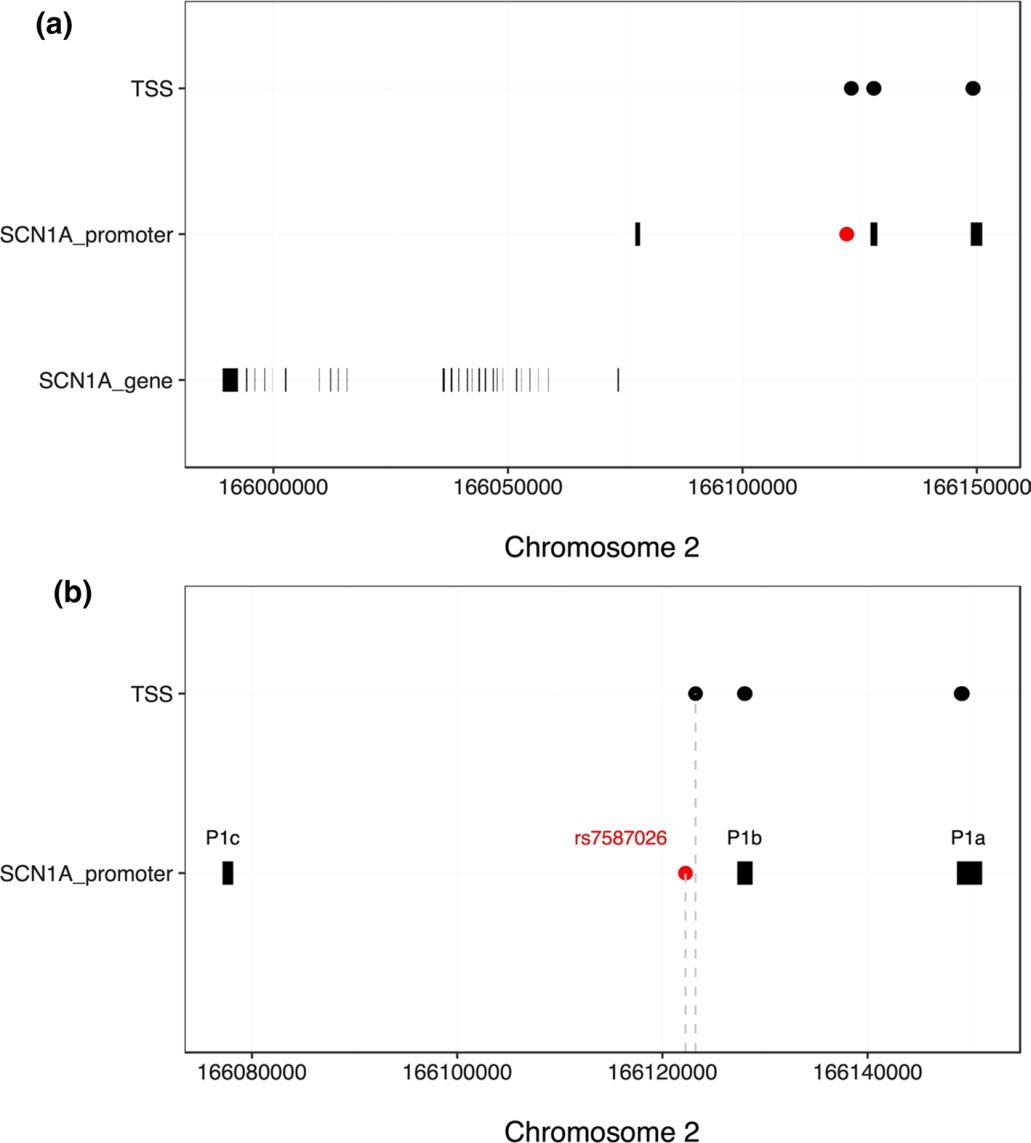
**a** Transcription start sites (TSSs) identified by the FANTOM5 project, located upstream of the *SCN1A* gene body. Six TSSs fell within two of the three *SCN1A* promoters and one TSS was located 981 bp away from rs7587026. **b** X-axis: genetic positions on chromosome 2, Y-axis: genetic elements that were considered

The FANTOM5 CAGE data set identified seven transcription start sites (TSS) located upstream of the *SCN1A* gene body: three mapped to the P1a promoter, three to the P1b promoter, and one closer to rs7587026 (distance: 981 bp) (Fig. 5). The six TSSs falling in the *SCN1A* promoters were recognised as *SCN1A*-related TSSs by the FANTOM5 project. The TSS located 981 bp from rs7587026 was not identified as a TSS for *SCN1A* or any other gene.

**Fig. 6:**
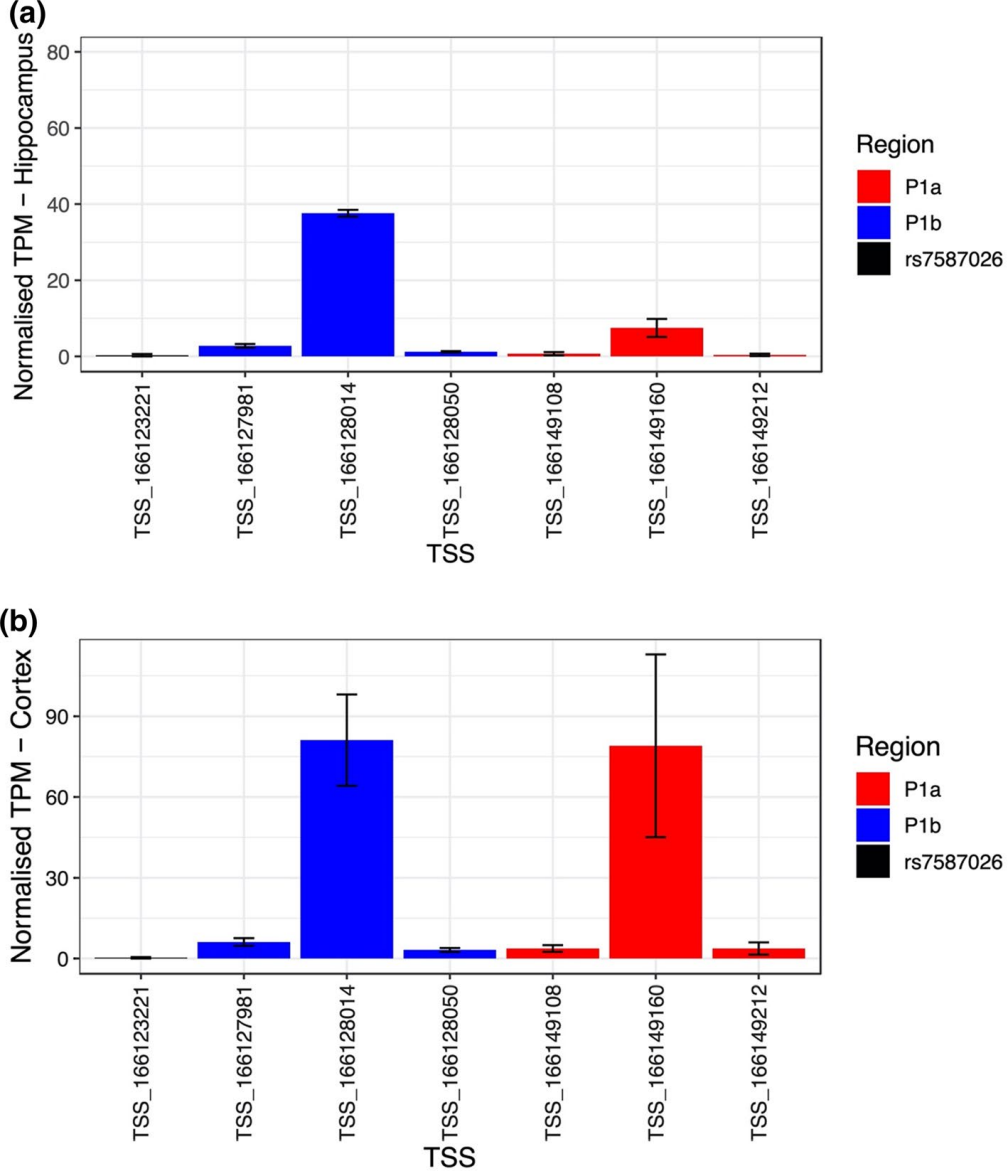
Normalized tags per million (TPM) expression values of the TSSs located upstream of *SCN1A* in the hippocampus (**a**) and cortex (**b**). In red are shown the TSSs located in P1a, in blue the TSSs in P1b and in black the TSS close to rs7587026. The black bars indicate the standard error (SE) of the mean

Considering the relative activity of the TSSs across the hippocampus and brain cortex, the TSS closest to rs7587026 was the least active in both tissues, accounting for a mean of 0.32 TPM in the hippocampus and 0.26 in the cortical samples, indicating that for every 1,000,000 CAGE tags in the CAGE library, an average of 0.32 in hippocampus and 0.26 in the cortex originated from this TSS. In both hippocampus and cortex, TSS:166128014, in P1b, was the most active, accounting for a mean of 37.61 TPM in the hippocampal samples and 81.11 in the cortical samples. The second most active TSS was TSS:166149160, which accounted for a mean of 7.48 TPM in hippocampus and 79.04 in the cortex (Fig. 6).

### rs7587026-containing genomic motif regulates *SCN1A* expression

**Fig. 7:**
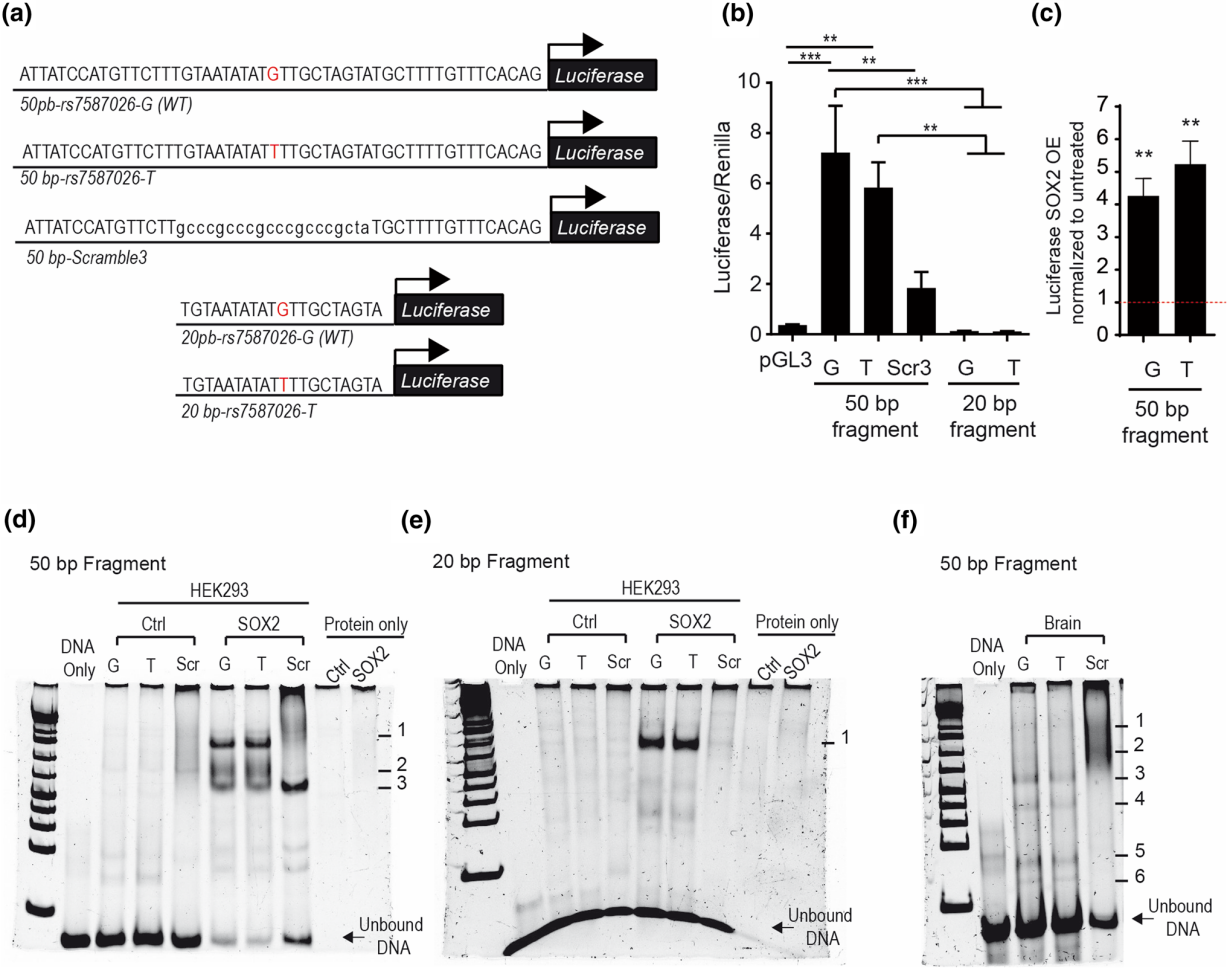
Genomic *SCN1A* fragment harbouring SNP rs7587026 has promoter activity. **a** DNA sequence and schematic representation of genomic fragments of *SCN1A* cloned in luciferase constructs. The 50 bp fragments are denoted as 50 bp-rs7587026-G (WT) (for the major allele/wild type sequence), 50 bp-rs7587026-T for the minor allele sequence and as 50 bp-Scramble3 (for the scramble sequence); the 20 bp fragments are denoted as 20 bp-rs7587026-G (WT) (for the major allele/wild type sequence) and 20 bp-rs7587026-T for the minor allele sequence. **b** Luciferase activity of NS20Y cells transfected with the 50 bp luciferase fragments with both genotypes and a scrambled sequence (constructs: *SCN1A*-50 bp-rs7587026-G(WT)-Luciferase, *SCN1A*-50 bp-rs7587026-T-Luciferase or *SCN1A*-50 bp-Scramble3-Luciferase) and 20 bp luciferase fragments of both genotypes (constructs: *SCN1A*-20 bp-rs7587026-G(WT)-Luciferase or *SCN1A*-20 bp-rs7587026-T-Luciferase). Luciferase values were normalized to Renilla values. Data is represented as mean ± SEM (one-way ANOVA, Tukey’s multiple comparison test, *n* = 4). **c** Luciferase activity of NS20Y cells co-transfected with the 50 bp-*SCN1A* fragments (*SCN1A*-50 bp-rs7587026-G(WT)-Luciferase or *SCN1A*-50 bp-rs7587026-T-Luciferase) and pCMV-Sox2-T2A-GFP (50 ng). Luciferase values were normalized to the untreated condition (data is represented as mean ± SEM, *n* = 4, One sample *t* test). **d**,**e** EMSA performed with HEK293T cells overexpressing pCMV-Sox2-T2A-GFP or hypB-CAG-2A-eGFP (control) with the 50 bp *SCN1A* fragments (**d**) and the 20 bp *SCN1A* fragment (**e**). Scr denotes scrambled sequences (sequences in Supplementary Table 2, online resource). **f** EMSA reaction with nuclear protein extracts from mouse brain extract and the 50 bp *SCN1A* fragments. Numbers denote DNA–protein complexes

After transfection of NS20Y cells with the luciferase constructs (Fig. 7a), we observed a dramatic increase of luciferase activity in the 50 bp fragments for both genotypes (G-major allele and T-minor allele) when compared to the pGL3 basic empty vector (Fig. 7b), showing that this fragment indeed contains transcription factor binding sites and can fundamentally activate gene transcription. However, we did not detect a difference in promoter activity between the two genotypes.

Luciferase activity measurements of NS20Y cells transfected with the three additional reporter constructs showed that the promoter activity was strongly reduced if the Sox2 binding site was destroyed (Scr3, Fig. 7b), and even completely abolished if the flanking sequences were deleted (20 bp fragment; Fig. 7b), suggesting that Sox2 plays a role in mediating transcriptional activation, but is not solely sufficient.

To further examine if Sox2 on its own has the potential to activate the transcriptional activity of the 50 bp genomic *SCN1A* fragment, we co-transfected the reporter plasmids under transcriptional control of the 50 bp fragments (G-major allele and T-minor allele) together with an expression plasmid for Sox2 into NS20Y cells. We found that Sox2 increased luciferase activity when compared to the untreated condition (Fig. 7c; fold increase of 4.255 ± 0.5385, *P* = 0.0042 and 5.233 ± 0.7104, *P* = 0.0052 for *SCN1A*-50 bp-rs7587026-G(WT)-Luciferase and *SCN1A*-50 bp-rs7587026-T-Luciferase, respectively, *n* = 4, one sample *t* test). We did not observe a significant difference in activation between the two genotypes tested. Next, we investigated if Sox2 directly binds to the genomic *SCN1A* fragments and if there is a difference in binding efficiency between the two genotypes. To this end, we performed electrophoretic mobility shift assays (EMSA) with the 50 bp genomic *SCN1A* fragments and nuclear protein extracts derived from HEK293T cells transfected with CAG-eGFP (control) or pCMV-Sox2-T2A-GFP. Interestingly, we observed three bands corresponding to DNA–protein complexes between the 50 bp fragments of both genotypes (G-major allele and T-minor allele), which could only be detected if Sox2 was expressed (Fig. 7d). One of these DNA–protein complexes remained even if the site surrounding the SNP was mutated (scrambled condition, scr), indicating that Sox2 can also bind to the flanking sequence. When the 20 bp genomic *SCN1A* fragments were subjected to an EMSA with nuclear extract from HEK293 cells expressing Sox2, only one band remained (Fig. 7e). Together, these results suggest that the sequence surrounding the SNP can bind to Sox2, as suggested by bioinformatic data given above; there does not appear to be a significant difference in binding efficiency between the genotypes. However, our findings also indicate that Sox2 can also bind to sites outside of this region. Finally, we examined if the genomic 50 bp *SCN1A* fragment containing the SNP binds to proteins present in nuclear extracts prepared from mouse brain (Fig. 7f). EMSA showed that multiple defined complexes formed for both genotypes. The pattern for the scrambled oligonucleotide strongly differed from the observed native band patterns, suggesting that the region around the SNP defines which transcription factors bind to this sequence. Additional bioinformatic analyses using the JASPAR database with a stringent cut off of 90% predicted that additional transcription factors (Supplementary Table 8, online resource) could bind to the 50 bp-*SCN1A* fragment both within the area surrounding the SNP (e.g., *FOXL1* and *RFX7*) as well as the flanking sequences (e.g., *GATA2*).

## Discussion

MTLEHS is a drug-resistant epilepsy syndrome of unknown—and likely multifactorial—causation [51, 53, 57]. A significant proportion of patients with MTLEHS have a history of FS [21, 45]. We previously reported a genetic association between MTLEHS + FS and rs7587026 [29]. The current findings suggest that the association is mediated by the consequences of regionally increased *SCN1A* expression related to the risk variant.

Between incident FS and onset of habitual seizures in MTLEHS + FS, there is a ‘latent period’, during which epileptogenesis is believed to occur [21, 62], as supported by data from animal models [36]. The period represents a potential window of opportunity to interfere with processes of epileptogenesis and neurodegeneration [41, 54]; it is a major area of research interest in developing anti-epileptogenesis strategies [41]. In humans, intervention would require both identification of which children are at risk of developing MTLEHS after FS and an effective anti-epileptogenic therapeutic strategy based on a mechanistic understanding.

The possibility that, in people who go on to develop MTLEHS after FS, the hippocampus may have been ‘vulnerable’ to FS/febrile status-induced damage has long been debated [3, 50]. If correct, demonstration of pre-FS hippocampal abnormalities might identify children at risk of developing MTLEHS after FS. There are no published MRI studies showing underlying pre-FS structural brain abnormality in children who have FS and then go on to have MTLEHS. Acute MRI post-febrile seizures, particularly post-febrile status, has shown hippocampal swelling and T2 hyperintensity that may in some cases proceed to the appearances of HS [37]. Underlying hippocampal ‘vulnerability’ is supported by the FEBSTAT study: compared to children with simple FS, children with febrile status showed, at baseline after febrile seizures, reduced hippocampal volumes and reversed right/left hippocampal volume ratios, even in the absence of signs of acute hippocampal damage [37]. However, as measuring pre-FS hippocampal volumes in healthy children is not feasible, an alternative strategy is needed to identify children who may be at risk following FS.

Genetics offers one possible route to identifying at risk individuals. FS are amongst the most heritable type of seizure [32]. Feenstra et al. identified a total of six SNPs associated with FS after measles, mumps, and rubella (MMR) vaccination (*IFI44L* rs273259; *CD46* rs1318653), or non-MMR linked FS in general (*SCN1A* rs6432860; *SCN2A* rs3769955; *ANO3* rs114444506; 12q21.33 rs1110546), but they also provided evidence that the six SNPs were not associated with post-FS epilepsy [15]. We previously showed that common variation in *SCN1A* at rs7587026 is associated specifically with MTLEHS + FS [29]. rs7587026 was not associated with FS in general—either in our study, or in the Feenstra study [15]—suggesting that there are genetically distinct susceptibilities to FS in general and to FS that are specifically associated with subsequent epilepsy.

It is not feasible to conduct large-scale MRI studies in healthy children to directly address the hypothesis that in children who experience FS and go on to develop MTLEHS, the hippocampus is structurally vulnerable to FS-induced injury. In adults with epilepsies, secondary processes, such as seizure-related neurodegeneration, may be confounding factors; for example subcortical volume differences seen on MRI in mesial temporal lobe epilepsy are correlated with disease duration [61]. Therefore, to determine whether rs7587026, with its demonstrated consequences on *SCN1A* expression in disease, affects brain structure, we examined healthy humans using the QTIM cohort. We show that rs7587026 is robustly associated with smaller hippocampal volume, in support of the idea that individuals at risk of MTLEHS after FS may have hippocampi with pre-existing vulnerability [3, 50]. Amygdala and thalamic volumes are also reduced in healthy minor allele homozygotes, suggesting that structures other than the hippocampus may also have pre-existing vulnerability upon which disease may act. We note that age does not have a differential effect on these subcortical volumes by minor allele homozygosity (Supplementary Fig. 4 and Supplementary Table 7, online resource).

We show that homozygosity for the risk variant has further functional consequences that may underpin disease susceptibility. In the human hippocampus, rs7587026 is associated with increased *SCN1A* expression (which is approximately doubled). The effect is specific for this SNP, and is not seen with unlinked common variants in *SCN1A*, including the *SCN1A* SNP (rs6432860) associated with FS in general identified by Feenstra et al. [15]. Addressing the possibility that this observed consequence is an artefact of HS severity affecting the number of cells available to express *SCN1A*, we show that rs7587026 is not associated with severity of HS/cell loss in resected sclerosed hippocampi from people with MTLEHS.

An alternative to the idea that FS leading to MTLEHS are a marker of underlying hippocampal vulnerability is that they might directly cause hippocampal injury and epileptogenesis. In a murine model for pleiotropic *SCN1A*-related epilepsy (*Scn1a*^*RH*±^), both prolonged [13] and short [48] exposure to hyperthermia-induced seizures precipitated development of a more severe phenotype than in those without exposure, or in exposed wildtype mice [48], suggesting that febrile seizures and *Scn1a* haploinsufficiency interact to produce a severe phenotype [48]. MTLEHS + FS involves an analogous possible precipitating event. As opposed to haploinsufficiency, however, we show that there is an association between rs7587026 genotype and *SCN1A* expression levels. Contrasting under- and over-expression directly is likely too simplistic and further understanding of regional and temporal patterns of expression, at cellular levels during development, and how these might affect neurophysiological properties at a network level, is required. For example, common genetic variants associated with brain surface area identified in a GWAS meta-analysis were enriched for regulatory elements active in the mid-foetal period [22]. In any case, based on the localised overexpression of *SCN1A* in the epileptogenic foci in adult brain, and smaller hippocampal volumes in healthy adult brain, we hypothesised that overexpression of *SCN1A* is causally related to increased seizure susceptibility.

To test this hypothesis, we generated a new zebrafish *scn1lab* overexpression model. Most (84%) *scn1lab-*OE larvae exhibited spontaneous seizures as measured by EEG. Moreover, *scn1lab-*OE zebrafish larvae had heightened incidence of temperature-induced convulsions. As expected, sodium channel blockers oxcarbazepine and valproic acid successfully repressed electric seizure activity; that the sodium-channel blocker phenytoin did not may be a species-dependent phenomenon [2], perhaps due to lack of absorption and/or rapid metabolism and excretion in larval zebrafish. Overall, our results provide functional evidence for a thermosensitive seizure-promoting effect of *scn1a* overexpression.

We were unable to determine a definite route for the association of rs7587026 on *SCN1A* transcription, leading us to seek alternative explanations for its prospective functional relevance. Our in silico analysis did not suggest a protein-coding function for the SNP. According to the most up to date *SCN1A* annotation, rs7587026 is located in an intronic region between the P1b and P1c *SCN1A* promoters. Using the map of sequence constraint as an indicator of the likelihood of functionality, rs7587026 lies within a highly constrained region of the human genome, suggesting that this region might be functionally relevant. The FANTOM5 CAGE data set also identified, close to rs7587026, a TSS peak not identified as a TTT for *SCN1A* or any other gene. While the lack of RNAseq supported introns in the region suggests that transcription here does not generate an alternative transcript of SCN1A, this signal could indicate an enhancer. Together, these observations led us to hypothesise that rs7587026 falls within a regulatory element that may operate in brain development; such epigenetic regulation may also explain regional specificity of the association between rs7587026 genotype and *SCN1A* expression [20].

To test this hypothesis, we performed several complementary in vitro analyses. We confirmed that a short genomic fragment in the *SCN1A* gene surrounding rs7587026 has intriguingly strong transcriptional activity and is able to bind, as predicted by the bioinformatic analysis (see Supplementary Material in online resource), to the transcription factor SOX2; the short genomic motif, furthermore, can be bound in vivo by a striking multitude of transcription factors. These data underline the biological complexity and relevance of transcriptional regulation involving rs7587026. In this simplified in vitro system, our analyses did not reveal a difference in the promoter activity or SOX2 binding affinity between the two rs7587026 genotypes. Several reasons can account for this discrepancy between the in vitro data and our complementary experiments on rs7587026 effects on *SCN1A* transcript abundance under in vivo conditions in human brain biopsies: given the obvious regulatory complexity of this novel promoter motif for *SCN1A*, additional transcription factors, transcription factor complexes, trans-regulatory promoter regulation as well as expression quantitative trait loci (eQTLs) may contribute to the observed differential expression of *SCN1A* in brain biopsy tissue of patients with epilepsy stratified according to the rs7587026 genotype, as compared to the distilled in vitro expression test system we used. In addition, one or more transcription factors that binds to the sequence may only be up-regulated in the diseased brain. The present in vitro experiments clearly indicate a strong regulatory role for *SCN1A* expression of the rs7587026-containing genomic motif, even if we were unable to define the precise mechanism (it is typically not possible to entirely replicate the complexity of in vivo promoter regulation in in vitro systems): in this context, the rs7587026 variant has a definite relevance, as our corresponding data from human epileptogenic brain tissue and MRI from healthy individuals clearly demonstrate. Whilst we note that rs7587026 and the P1b *SCN1A* promoter fall in the same linkage disequilibrium block, the experimental results presented here suggest that a short genomic fragment around rs7587026 alone has promoter activity independent of P1b, located further away in the same linkage disequilibrium block. Finally, we note that the JASPAR and ReMap resources produced contradictory in silico results: these resources were created using different input data and pipelines (JASPAR used internally produced data, HT-SELEX, PBMs, and ChIP-seq and DAP-seq experiments from CistromeDB, ReMap 2020 [ReMap 2020 itself used data from GEO, ENCODE, ENA], ChIP-atlas and ModERN, whilst ReMap (2022) used GEO, ArrayExpress, ENCODE). The discrepancies are, therefore, not necessarily surprising and demonstrate the importance of further empirical data, which we provide, and an overarching perspective.

It remains unknown how increased *SCN1A* expression leads to reduced hippocampal volume. The generalisability to children of our human histopathology and imaging data, which were obtained from adults, is unproven. However, showing that genotypically driven increased *SCN1A* expression reduces hippocampal volume before the occurrence of FS in people (children) who go on to develop MTLEHS would face insurmountable ethical and logistic implications (for example, MRI at this age would have to be conducted under general anaesthesia). Our approach provides new insights into the association between FS and MTLEHS that would otherwise be difficult to obtain.

Our studies of *SCN1A* expression in hippocampi of individuals of different rs7587026 type, and of patterns of neuronal loss, is limited by the small numbers of minor allele homozygotes, influenced by the minor allele frequency of 0.302 [29], and the rarity of hippocampal specimens available for study. For the study of neuronal loss, we addressed this limitation by performing a replication study in a separate set of samples (Supplementary Fig. 3, online resource). Unfortunately, this was not possible for the *SCN1A* expression data.

In conclusion, having previously shown that rs7587026 is associated with increased susceptibility to MTLEHS + FS, we demonstrate a potential mechanistic link through increased hippocampal *SCN1A* expression, smaller hippocampal volumes and an increased propensity to temperature-induced seizures in a model overexpressing *scn1a*.

There is a need for biomarkers of epileptogenesis [14]. Our results suggest that rs7587026 could become a biomarker in children for those at risk of developing epilepsy after FS, opening up the possibility of its use in studies aiming to identify anti-epileptogenic treatments after such insults. Moreover, the consequences we have shown of increased *Scn1a* expression point to the need for quantitative precision in genetic therapies intended to increase *SCN1A* transcription [10] for treatment of seizures in Dravet syndrome associated with *SCN1A* haploinsufficiency.

## Supplementary Information

Below is the link to the electronic supplementary material.

Supplementary file1 (DOCX 12986 kb)

## Data Availability

Data that support the findings of this study are available from the corresponding author upon reasonable request and subject to requirements of the sources for individual components of the study.

## Declarations

### Research involves human or animals participants

All procedures performed in studies involving human participants were in accordance with the ethical standards of the institutional and/or national research committee and with the 1964 Helsinki declaration and its later amendments or comparable ethical standards. All experiments involving zebrafish were performed in compliance with the European Community Council Directive of November 2010 for Care and Use of Laboratory Animals (Directive 2010/63/EU), and the ARRIVE guidelines. The Norwegian Food Safety Authority via its experimental animal administration’s supervisory and application system approved all animal experimentation (FOTS ID 15469 and 23935).
