# Supplementary material for: SCN1A overexpression, associated with a genomic region marked by a risk variant for a common epilepsy, raises seizure susceptibility
Source: Acta Neuropathol. 2022 May 12;144(1):107–27. doi: 10.1007/s00401-022-02429-0 (PMC9217876; doi:10.1007/s00401-022-02429-0)
Supplement: Supplementary file 1 — Supplementary file1 (DOCX 12986 kb) [file 401_2022_2429_MOESM1_ESM.docx]

Supplementary Information to

*SCN1A* overexpression, associated with a genomic region marked by a risk variant for a common epilepsy, raises seizure susceptibility

Katri Silvennoinen,^1,2†^ Kinga Gawel,^3,4†^, Despina Tsortouktzidis,^5,6†^ Julika Pitsch,^5,6^ Saud Alhusaini,^7,8^ Karen M. J. van Loo,^5,9^ Richard Picardo,^10^ Zuzanna Michalak,^10^ Susanna Pagni, ^1,2^ Helena Martins Custodio, ^1,2^ James Mills, ^1,2^ Christopher D. Whelan,^7,11^ Greig I. de Zubicaray,^12^ Katie L. McMahon,^13^ Wietske van der Ent,^3^ Karolina J. Kirstein-Smardzewska,^3^ Ettore Tiraboschi,^3^ Jonathan M. Mudge,^14^ Adam Frankish,^14^ Maria Thom,^10^ Margaret J. Wright,^15^ Paul M. Thompson,^11^ Susanne Schoch,^5,6^ Albert J. Becker,^5*^ Camila V Esguerra^3*^ and Sanjay M. Sisodiya^1,2*^

^†^**These authors contributed equally to this work.**

^*^ **These authors contributed equally to this work.**

1 Department of Clinical and Experimental Epilepsy, UCL Queen Square Institute of Neurology, London, WC1N 3BG, UK

2 Chalfont Centre for Epilepsy, Chalfont St Peter, Bucks, SL9 0RJ, UK

3 Chemical Neuroscience Group, Centre for Molecular Medicine Norway (NCMM), Faculty of Medicine, University of Oslo, 0349 Oslo, Norway

4 Department of Experimental and Clinical Pharmacology, Medical University of Lublin, 20-090 Lublin, Poland

5 Section for Translational Epilepsy Research, Department of Neuropathology, University Hospital Bonn, 53127 Bonn, Germany

6 Department of Epileptology, University Hospital Bonn, 53127 Bonn, Germany

7 Department of Molecular and Cellular Therapeutics, The Royal College of Surgeons in Ireland, Dublin 2, Ireland.

8 Department of Neurology, Yale University School of Medicine, New Haven, CT 06520, USA

9 Department of Epileptology, Neurology, RWTH Aachen University, 52074 Aachen, Germany

10 Department of Neuropathology, UCL Queen Square Institute of Neurology, London, WC1N 3BG, UK

11 Imaging Genetics Center, Mark and Mary Stevens Neuroimaging and Informatics Institute, Keck School of Medicine, University of Southern California, Marina del Rey, CA 90292, USA

12 School of Psychology, Faculty of Health, Queensland University of Technology (QUT), Brisbane, QLD 4059, Australia

13 School of Clinical Sciences, Faculty of Health, Queensland University of Technology (QUT), Brisbane, QLD 4029, Australia

14 European Molecular Biology Laboratory, European Bioinformatics Institute, Wellcome Genome Campus, Cambridge, CB10 1SD, UK

15 Queensland Brain Institute, University of Queensland, St Lucia, 4072 QLD, Australia

*Corresponding author:

Sanjay M Sisodiya

Department of Clinical and Experimental Epilepsy, UCL Queen Square Institute of Neurology, Box 29, Queen Square, London WC1N 3BG, United Kingdom

Email: s.sisodiya@ucl.ac.uk

Tel: +44 2034488612

**CONTENTS**

Supplementary methods 3

1. Neuroimaging 3

2. Promoter analysis 3

Supplementary Table 1 3

Supplementary Table 2 4

Supplementary results

1*. SCN1A* mRNA expression levels in hippocampi of patients with 5

pharmacoresistant TLE

Supplementary Table 3 5

Supplementary Figure 1 5

Supplementary Figure 2 6

2. Hippocampal staining by subfield and rs7587026 type 7

Supplementary Figure 3 7

Supplementary Table 4 8

Supplementary Table 5 8

3. Neuroimaging 9

Supplementary Table 6 9

Supplementary Figure 4 10

Supplementary Table 7 11

4. Bioinformatics 12

5. Promoter analysis 12

Supplementary Table 8 12

Supplementary references 13

**SUPPLEMENTARY METHODS**

**1. Neuroimaging**

QTIM inclusion criteria

All participants were screened (by self‐report) for their suitability for any significant medical, psychiatric, neurological conditions (including head injuries), current or past diagnosis of substance abuse and for current use of psychoactive medication. All individuals were of European ancestry and right‐handed as assessed by the Annett's Handedness Questionnaire.[1] Zygosity of same‐sex twins was established by DNA typing of nine markers (AmpF1STR Profiler Plus Amplification KIT, Applied Biosystems Inc., Foster City, CA, USA) and later confirmed for >80% of the sample who were genotyped on the Illumina 610K SNP array.

**2. Promoter analysis**

**Supplementary Table 1. Primers for cloning**

| **Construct name** | **Oligonucleotides sequence 5´-3´** |
| --- | --- |
| *SCN1A-50bp-rs7587026-G(WT)-Luciferase/*  *SCN1A-50bp-rs7587026-T-Luciferase* | forward: tcttacgcgtgctagattatccatgttctttgtaatata reverse: cttagatcgcagatcctgtgaaacaaaagcatactagca |
| *SCN1A-50bp-Scramble3-Luciferase* | forward:ctagattatccatgttcttgcccgcccgcccgcccgctatgcttttgtttcacag reverse:gatcctgtgaaacaaaagcatagcgggcgggcgggcgggcaagaacatggataat |
| *SCN1A-20bp-rs7587026-G(WT)-Luciferase* | forward: ctagtgtaatatatgttgctagta  reverse: gatctactagcaacatatattaca |
| *SCN1A-20bp-rs7587026-T-Luciferase* | forward: ctagtgtaatatattttgctagta  reverse: gatctactagcaaaatatattaca |

**Supplementary Table 2. oligonucleotides for EMSA**

| **Oligonucleotides name** | **Oligonucleotides sequence 5´-3´** |
| --- | --- |
| *SCN1A-50bp-rs7587026-G(WT)* | forward: attatccatgttctttgtaatatatgttgctagtatgcttttgtttcacag reverse: ctgtgaaacaaaagcatactagcaacatatattacaaagaacatggataat |
| *SCN1A-50bp-rs7587026-T* | forward:attatccatgttctttgtaatatattttgctagtatgcttttgtttcacag reverse:ctgtgaaacaaaagcatactagcaaaatatattacaaagaacatggataat |
| *SCN1A-50bp-Scramble* | forward: attatccatgttcttcccccccccccccccccccctgcttttgtttcacag reverse:ctgtgaaacaaaagcaggggggggggggggggggggaagaacatggataat |
| *SCN1A-20bp-rs7587026-G(WT)* | forward: tgtaatatatgttgctagta  reverse: tactagcaacatatattaca |
| *SCN1A-20bp-rs7587026-T* | forward: tgtaatatattttgctagta  reverse: tactagcaaaatatattaca |
| *SCN1A-20bp-Scramble2* | forward: cccccccccccccccccccc  reverse: gggggggggggggggggggg |

**SUPPLEMENTARY RESULTS**

**Supplementary Table 3: *SCN1A* mRNA expression levels values in hippocampi of patients with pharmacoresistant MTLE**

| **Genotype** | **Median** | **Mean** | **Range** | **Standard deviation** |
| --- | --- | --- | --- | --- |
| **rs7587026 CC** | 10.49 | 12.06 | 1.22-37.56 | 7.80 |
| **rs7587026 AC** | 13.75 | 14.26 | 2.33-31.02 | 7.95 |
| **rs7587026 AA** | 23.34 | 25.54 | 7.52-48.49 | 11.92 |
| **rs922224 AA** | 15.20 | 15.62 | 1.36-35.20 | 7.97 |
| **rs922224 AG** | 10.26 | 13.91 | 1.22-48.49 | 10.15 |
| **rs922224 GG** | 10.72 | 11.51 | 4.57- 22.05 | 6.11 |

**Supplementary Figure 1: Violin plots showing the expression levels of *SCN1A* in hippocampi of MTLEHS by SNP rs643280 genotype.** For SNP rs643280, no differences in *SCN1A* expression levels were observed for MTLEHS stratified by SNP genotype (MTLEHS: GG: n=40, GA: n=28, AA: n=6; Kruskal-Wallis test: *P*=0.325).

**
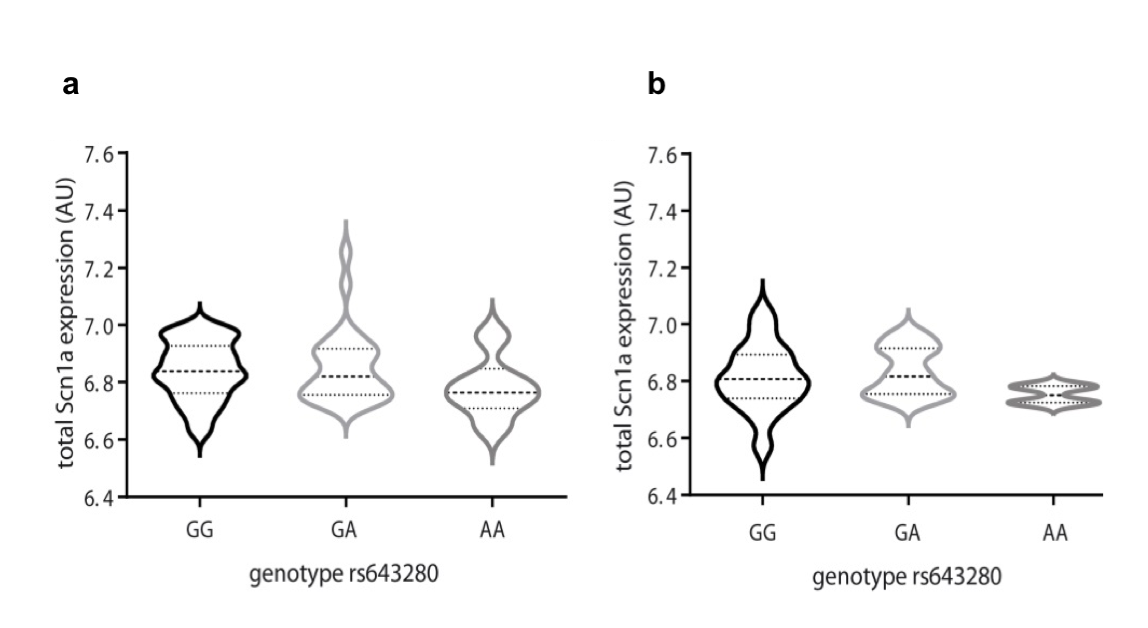
**

**Supplementary Figure 2: Genotype correlation of SNP rs7587026 with severity of neuronal cell loss in different hippocampal subfields.** No significant differences are present in biopsy specimen from patients with respect to allelic variants (CC: n=33, CA: n=33, AA: n=7; Kruskal-Wallis test: CA1: *P*=0.770, CA2: *P*=0.276, CA3: *P*=0.273, CA4: *P*=0.748). Neuronal loss: 3 – severe, 2 – moderate, 1- mild, 0 – no neuronal loss.


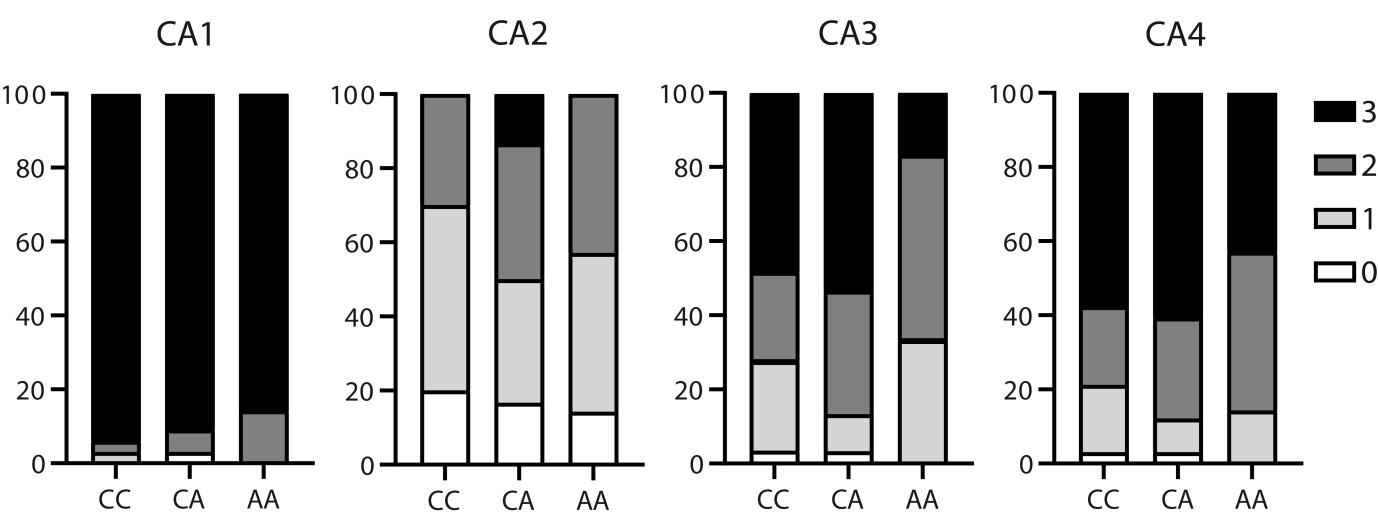


**2. Hippocampal staining by subfield and rs7587026 type**

**Supplementary Figure 3: Quantitative evaluation of the percentage staining in each hippocampal subfield by rs7587026 genotype in the replication cohort.** (a) NeuN and (b) MAP2 immunolabelling. The boxes encompass 25th to 75th percentiles with median shown as horizontal bar. The whiskers span minimum to maximum values. No significant differences in staining distribution by genotype for subfield were identified. See Supplementary Tables 1 and 2 for further details.

**
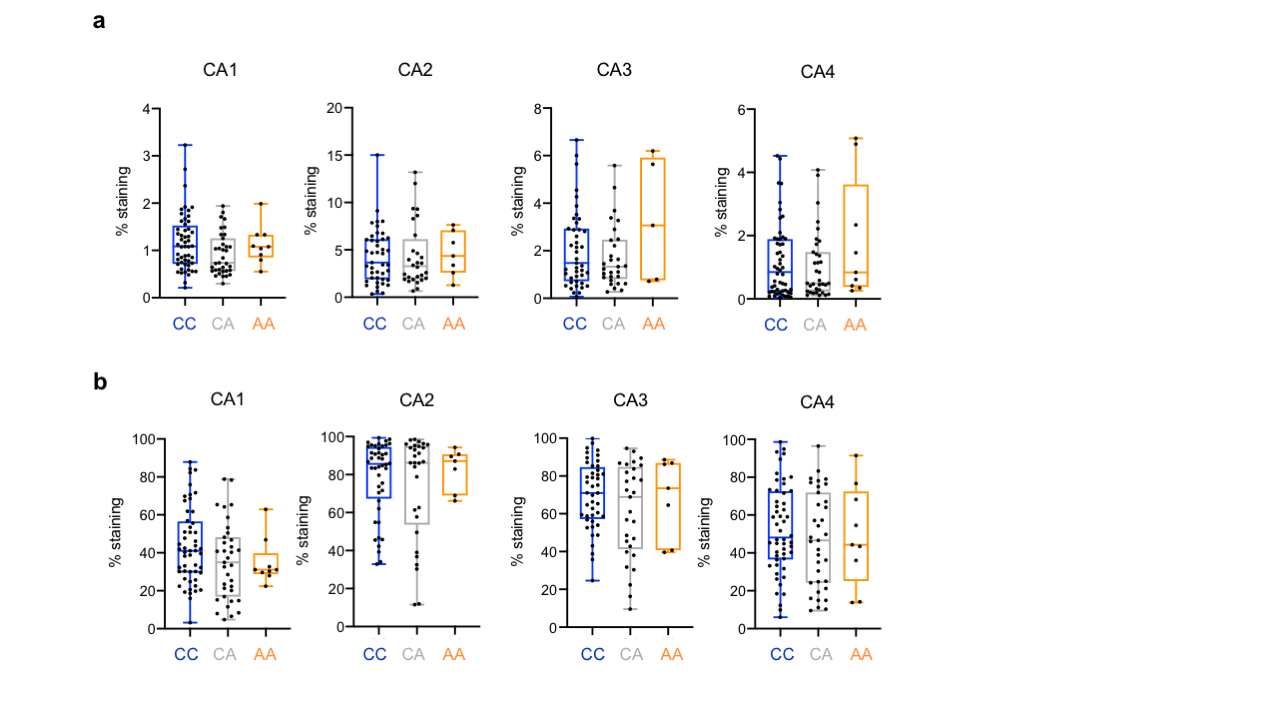
**

**Supplementary Table 4: Quantitative evaluation of percentage staining with NeuN in each hippocampal subfield by rs7587026 type.**

|  | **CA1** | | | **CA2** | | | **CA3** | | | **CA4** | | |
| --- | --- | --- | --- | --- | --- | --- | --- | --- | --- | --- | --- | --- |
|  | AA | AC | CC | AA | AC | CC | AA | AC | CC | AA | AC | CC |
| N | 9 | 35 | 52 | 7 | 28 | 43 | 5 | 29 | 43 | 9 | 35 | 52 |
| Median % staining | 1.08 | 0.74 | 1.09 | 4.36 | 3.28 | 3.65 | 3.06 | 1.33 | 1.48 | 0.84 | 0.49 | 0.85 |
| IQR | 0.48 | 0.69 | 0.81 | 4.48 | 4.21 | 4.23 | 5.16 | 1.65 | 2.21 | 3.24 | 1.24 | 1.67 |
| Comparison of distributions  (Mann-Whitney U) | AA vs AC  U=112, *P*=0.194 | | | AA vs AC  U=84, *P*=0.586 | | | AA vs AC  U=50, *P*=0.295 | | | AA vs AC  U=119, *P*=0.274 | | |
|  | AA vs CC  U=233*, P*= 0.984 | | | AA vs CC  U=136*, P*=0.702 | | | AA vs CC  U=78*, P*=0.338 | | | AA vs CC  U=184*, P*=0.309 | | |

**Supplementary Table 5: Quantitative evaluation of percentage staining with MAP2 in each hippocampal subfield by rs7587026 type.**

|  | **CA1** | | | **CA2** | | | **CA3** | | | **CA4** | | |
| --- | --- | --- | --- | --- | --- | --- | --- | --- | --- | --- | --- | --- |
|  | AA | AC | CC | AA | AC | CC | AA | AC | CC | AA | AC | CC |
| N | 9 | 35 | 52 | 7 | 29 | 44 | 7 | 29 | 44 | 9 | 35 | 52 |
| Median % staining | 31.1 | 34.9 | 41.1 | 87.2 | 86.2 | 85.6 | 73.5 | 68.9 | 71.0 | 44.3 | 46.6 | 48.1 |
| IQR | 11.0 | 31.3 | 27.0 | 21.7 | 41.4 | 27.3 | 46.1 | 68.9 | 27.5 | 47.4 | 47.6 | 36.0 |
| Comparison of distributions  (Mann-Whitney U) | AA vs AC  *P*=1.000 | | | AA vs AC  *P*=1.000 | | | AA vs AC  *P*=0.505 | | | AA vs AC  *P*=0.932 | | |
|  | AA vs CC  *P*= 0.238 | | | AA vs CC  *P*=0.904 | | | AA vs CC  *P*=1.000 | | | AA vs CC  *P*=0.684 | | |

**3. Neuroimaging**

**Supplementary Table 6. Demographics of the healthy participants included in the neuroimaging analyses by rs7587026 type.**

|  | **AA** | **AC** | **CC** | **AA vs. AC** | **AA vs. CC** | **AC vs. CC** |
| --- | --- | --- | --- | --- | --- | --- |
| Number (%) | 41 (6.9%) | 242 (40.5%) | 314 (52.6%) |  |  |  |
| Gender (%)  Male  Female | 15 (36.6%)  26 (63.4%) | 97 (40.1%)  145 (59.9%) | 124 (39.1%)  190 (60.5 %) | Pearson's  χ^2^=0.06    *P*=0.80 | Pearson's χ^2^=0.03    *P* =0.85 | Pearson's  χ^2^=0.002    *P* =0.96 |
| Age:  mean (SD) | 23.7 (3.46) | 23.8 (2.96) | 23.7 (3.20) | t=-0.07  *P* =0.94 | t=0.82  *P* =0.41 | t=1.93  *P* =0.05 |
| Zygosity (%)  Dizygotic  Monozygotic ^+^  None ^++^ | 18 (44%)  10 (24%)  13 (32%) | 121 (50%)  39 (16%)  82 (34%) | 141 (45%)  50 (16%)  123 (39%) | Pearson's χ^2^=1.70    *P* =0.42 | Pearson's χ^2^=2.07    *P* =0.35 | Pearson's  χ^2^=1.8    *P* =0.40 |

Abbreviations: SD – standard deviation

^+^ Single member of monozygotic twins included

^++^ Siblings

**Supplementary Figure 4:** **The correlation between age and total intracranial volume (ICV), and volume of subcortical structures.**

Data are presented for all participants (panel a), rs7587026 minor allele homozygotes (AA; n=41; panel b), rs7587026 minor allele heterozygotes (AC; n=242; panel c), and rs7587026 major allele homozygotes (CC; n= 314; panel d). Colour bar represents Pearson’s correlation coefficients, with positive correlations represented in blue and negative correlations represented in red. Circle sizes represent the degree of association. See Supplementary Table 4 for numerical values.

**
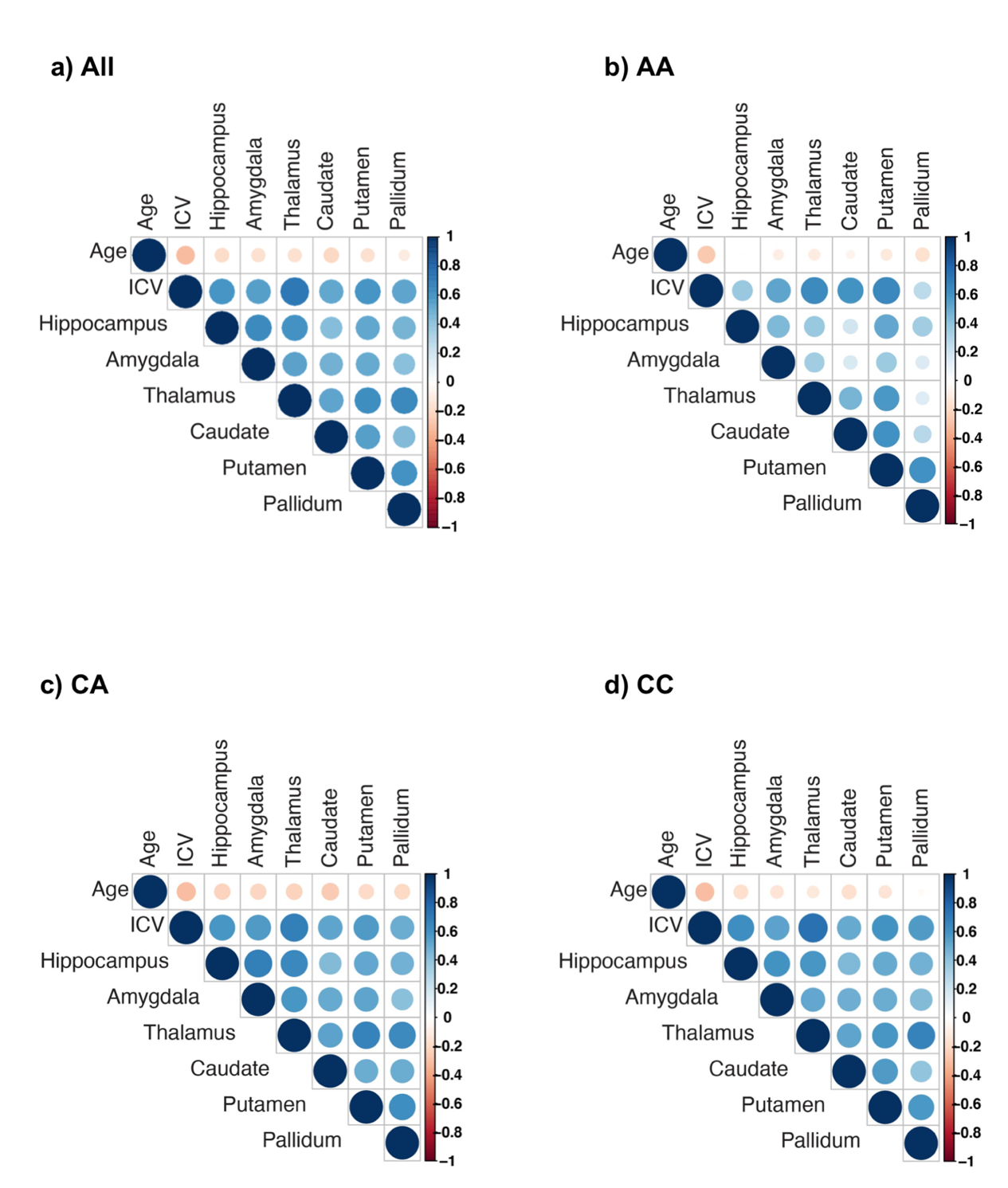
**

**Supplementary Table 7: Correlation between age and intracranial volume (ICV), and volume of subcortical structures.**

|  | **All individuals** | | **AA** | | **AC** | | **CC** | |
| --- | --- | --- | --- | --- | --- | --- | --- | --- |
|  | Pearson’s correlation coefficient | *P*  value | Pearson’s correlation coefficient | *P* value | Pearson’s correlation coefficient | *P* value | Pearson’s correlation coefficient | *P* value |
| ICV | -0.30 | 6.36 x 10^-14^ | -0.26 | 0.10 | -0.30 | 1.07 x 10^-6^ | -0.30 | 5.53 x 10^-8^ |
| Hippocampus | -0.18 | 8.73 x 10^-6^ | 0.007 | 0.96 | -0.22 | 0.0004 | -0.17 | 0.001 |
| Amygdala | -0.17 | 3.40 x 10^-5^ | -0.09 | 0.57 | -0.21 | 0.0006 | -0.15 | 0.008 |
| Thalamus | -0.17 | 4.36 x 10^-5^ | -0.11 | 0.50 | -0.22 | 0.0003 | -0.13 | 0.02 |
| Caudate | -0.20 | 4.80 x 10^-7^ | -0.06 | 0.70 | -0.26 | 4.48 x 10^-5^ | -0.18 | 0.001 |
| Putamen | -0.16 | 6.46 x 10^-5^ | -0.11 | 0.49 | -0.20 | 0.001 | -0.14 | 0.01 |
| Globus Pallidus | -0.10 | 0.01 | -0.17 | 0.30 | -0.21 | 0.001 | -0.03 | 0.63 |

**4. Bioinformatics**

Of potential interest is the intersection between rs7587026 and transcription-factor binding data. SOX2 has a fundamental role in the regulation of embryonic and adult stem cells [6], controlling cell fate decisions in a programmed manner via its cooperative binding with other transcription factors. The SOX2 binding region overlaps a ChIP-seq signal for FOXA1, which is known to be one such partner [5]; there is evidence that both proteins play a role in brain development, as well as in other developmental pathways. Pathogenic variants in *SOX2* can lead to hippocampal malformations in humans [7]. Meanwhile, the androgen receptor (AR) is a steroid-hormone activated transcription factor that mediates a range of physiological processes, including, but not limited to, the development of the male phenotype. Interestingly, it is well established that AR plays a core role in the modulation and structure of the hippocampus [2], while there is a general – although complex – relationship between hormones and epilepsy [8]. As defined by the JASPAR (2020) database [3], rs7587026 does not overlap any robust transcription factor binding sites, but a perfect androgen response element [AGAACA] ‘half-site’ [9] is found 12bp upstream in the JASPAR database. Altogether, although we are unable to provide a precise model for the observed consequences of rs7587026 at the present time, our data suggest it falls within a regulatory element that may operate in brain development; such epigenetic regulation may also explain regional specificity of the association between rs7587026 genotype and *SCN1A* expression [4].

**5. Promoter analysis**

**Supplementary Table 8. Prediction of transcription factors binding scores in the 50 bp-*SCN1A* fragment**

| **Matrix ID** | **Name** | **Relative score** | **Start** | **End** | **Strand** | **Predicted sequence** |
| --- | --- | --- | --- | --- | --- | --- |
| [MA0036.1](https://jaspar.genereg.net/matrix/MA0036.1) | MA0036.1.GATA2 | 1.000 | 3 | 7 | - | GGATA |
| [MA0442.1](https://jaspar.genereg.net/matrix/MA0442.1) | MA0442.1.SOX10 | 1.000 | 13 | 18 | + | CTTTGT |
| [MA0442.2](https://jaspar.genereg.net/matrix/MA0442.2) | MA0442.2.SOX10 | 0.942 | 11 | 21 | - | ATTACAAAGAA |
| [MA0098.1](https://jaspar.genereg.net/matrix/MA0098.1) | MA0098.1.ETS1 | 0.912 | 3 | 8 | + | TATCCA |
| [**MA0033.1**](https://jaspar.genereg.net/matrix/MA0033.1) | **MA0033.1.FOXL1** | **0.902** | **21** | **28** | **-** | **AAcATATA** |
| [MA0719.1](https://jaspar.genereg.net/matrix/MA0719.1) | MA0719.1.RHOXF1 | 0.960 | 1 | 8 | + | ATTATCCA |
| [MA1152.1](https://jaspar.genereg.net/matrix/MA1152.1) | MA1152.1.SOX15 | 0.958 | 38 | 47 | + | CTTTTGTTTC |
| [MA0867.2](https://jaspar.genereg.net/matrix/MA0867.2) | MA0867.2.SOX4 | 0.935 | 37 | 46 | - | AAACAAAAGC |
| [**MA1554.1**](https://jaspar.genereg.net/matrix/MA1554.1) | **MA1554.1.RFX7** | **0.927** | **25** | **33** | **+** | **TgTTGCTAG** |

*Prediction using JASPAR database, Input sequence: 50 bp-SCN1A (WT) fragment (cut off 90 %)*

*Lower case letters denote the position of the SNP in the WT sequence.*

**SUPPLEMENTARY REFERENCES**

1. Annett M (1970) A classification of hand preference by association analysis. Br J Psychol 61:303–321. https://doi.org/10.1111/j.2044-8295.1970.tb01248.x

2. Atwi S, McMahon D, Scharfman H, MacLusky NJ (2016) Androgen Modulation of Hippocampal Structure and Function. Neuroscientist 22:46–60. https://doi.org/10.1177/1073858414558065

3. Fornes O, Castro-Mondragon JA, Khan A, Van Der Lee R, Zhang X, Richmond PA et al (2020) JASPAR 2020: Update of the open-Access database of transcription factor binding profiles. Nucleic Acids Res 48:D87–D92. https://doi.org/10.1093/nar/gkz1001

4. Frankish A, Diekhans M, Ferreira AM, Johnson R, Jungreis I, Loveland J et al (2019) GENCODE reference annotation for the human and mouse genomes. Nucleic Acids Res 47:D766–D773. https://doi.org/10.1093/nar/gky955

5. Hagey DW, Klum S, Kurtsdotter I, Zaouter C, Topcic D, Andersson O et al (2018) SOX2 regulates common and specific stem cell features in the CNS and endoderm derived organs. PLoS Genet 14:e1007224. https://doi.org/10.1371/journal.pgen.1007224

6. Lodato MA, Ng CW, Wamstad JA, Cheng AW, Thai KK, Fraenkel E et al (2013) SOX2 Co-Occupies Distal Enhancer Elements with Distinct POU Factors in ESCs and NPCs to Specify Cell State. PLoS Genet 9:e1003288. https://doi.org/10.1371/journal.pgen.1003288

7. Sisodiya SM, Ragge NK, Cavalleri GL, Hever A, Lorenz B, Schneider A et al (2006) Role of SOX2 mutations in human hippocampal malformations and epilepsy. Epilepsia 47:534–542. https://doi.org/10.1111/j.1528-1167.2006.00464.x

8. Taubøll E, Sveberg L, Svalheim S (2015) Interactions between hormones and epilepsy. Seizure 28:3–11. https://doi.org/10.1016/j.seizure.2015.02.012

9. Wilson S, Qi J, Filipp F V (2016) Refinement of the androgen response element based on ChIP-Seq in androgen-insensitive and androgen-responsive prostate cancer cell lines. Sci Rep 6: 32611. https://doi.org/10.1038/srep32611
